# Supplementary material for: Unprecedented Elimination Reactions of Cyclic Aldols: A New Biosynthetic Pathway toward the Taiwaniaquinoid Skeleton
Source: Molecules. 2023 Feb 4;28(4):1524. doi: 10.3390/molecules28041524 (PMC9965844; doi:10.3390/molecules28041524)
Supplement: Supplementary file 1 [file molecules-28-01524-s001.zip › molecules-2163430-supplementary.pdf]

Supporting Information

Unprecedented Elimination Reactions of Cyclic Aldols: A New Biosynthetic Pathway toward the Taiwaniaquinoid Skeleton

Juan J. Guardia [1], Antonio Fernández [1], José Justicia [1], Houda Zentar [1], Ramón Alvarez- Manzaneda [2], Enrique Alvarez-Manzaneda [1] and Rachid Chahboun [1,*]

[1]*Departamento de Química Orgánica, Facultad de Ciencias, Instituto de Biotecnología, Universidad de Granada, 18071 Granada, Spain.*

[2]*Área de Química Orgánica, departamento de Química y Física, Universidad de Almería, 04120 Almería, Spain*

* *Correspondence: rachid@ugr.es*

Table of Contents

^{1}H and ^{13}C NMR Spectra 2-43

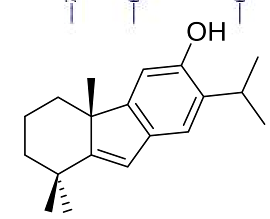

6

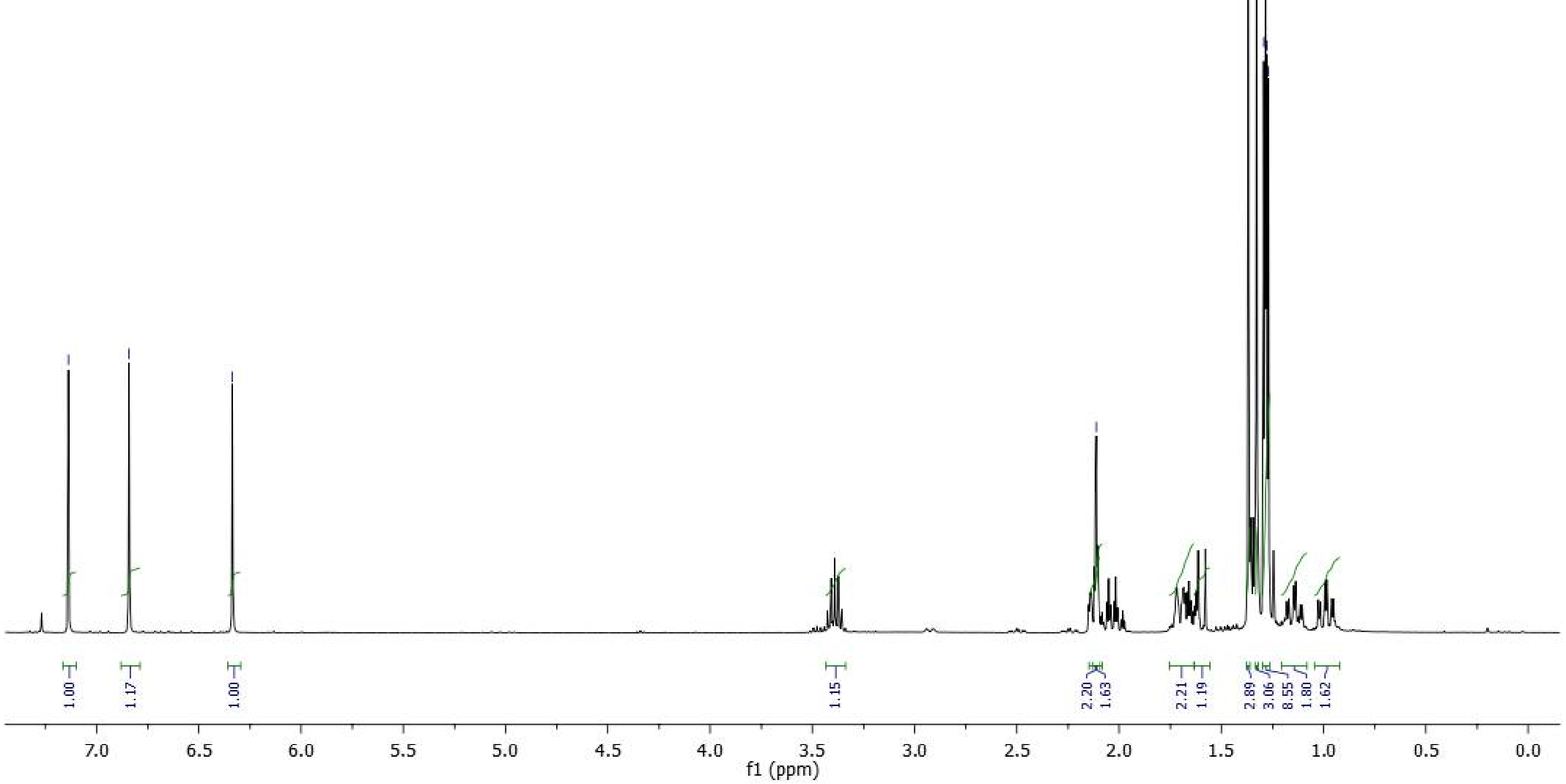
7.14
6.84
6.34
2.11
1.37
1.33
1.29
1.28
1.28
1.27
1.00
1.17
1.00
1.15
2.20
1.63
2.21
1.19
2.89
3.06
8.55
1.80
1.62
7.0
6.5
6.0
5.5
5.0
4.5
4.0
3.5
3.0
2.5
2.0
1.5
1.0
0.5
0.0
f1 (ppm)

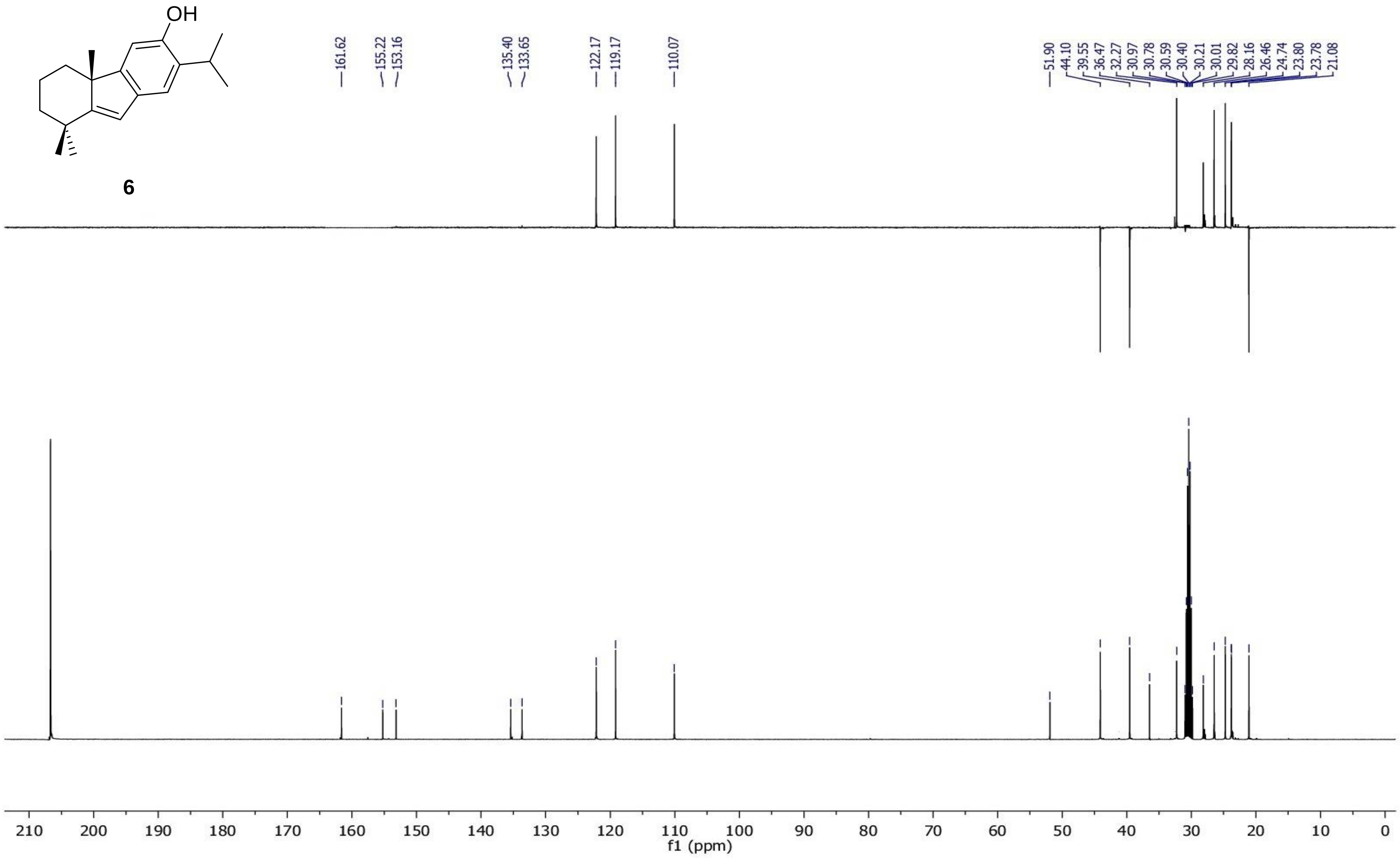
OH
6
161.62
155.22
153.16
135.40
133.65
122.17
119.17
110.07
51.90
44.10
39.55
36.47
32.27
30.97
30.78
30.59
30.40
30.21
30.01
29.82
28.16
26.46
24.74
23.80
23.78
21.08
210 200 190 180 170 160 150 140 130 120 110 100 90 80 70 60 50 40 30 20 10 0
f1 (ppm)

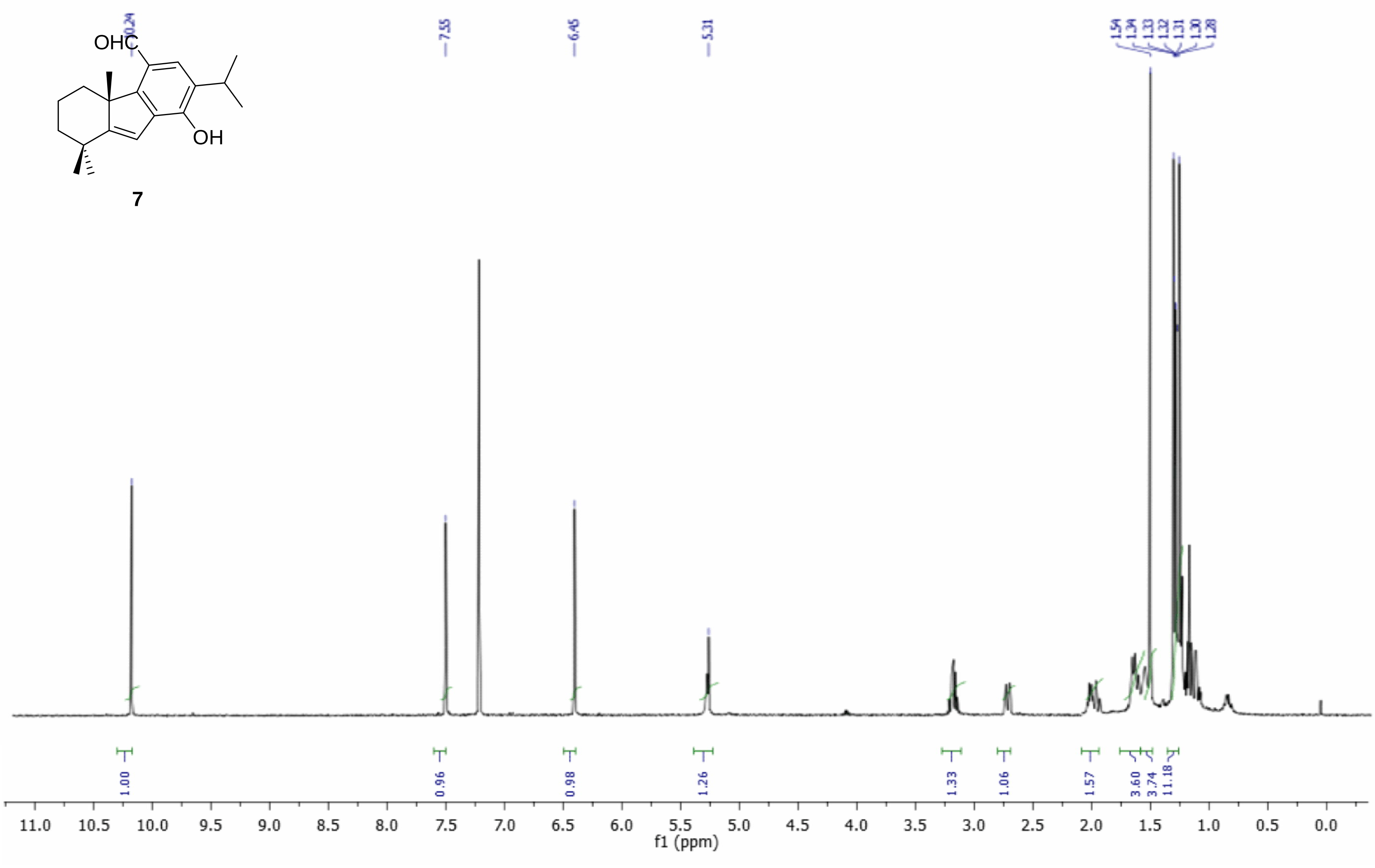
OHC
OH
7
10.24
7.55
6.45
5.31
1.54
1.34
1.33
1.32
1.31
1.30
1.28
1.00
0.96
0.98
1.26
1.33
1.06
1.57
3.60
3.74
11.18
11.0 10.5 10.0 9.5 9.0 8.5 8.0 7.5 7.0 6.5 6.0 5.5 5.0 4.5 4.0 3.5 3.0 2.5 2.0 1.5 1.0 0.5 0.0
f1 (ppm)

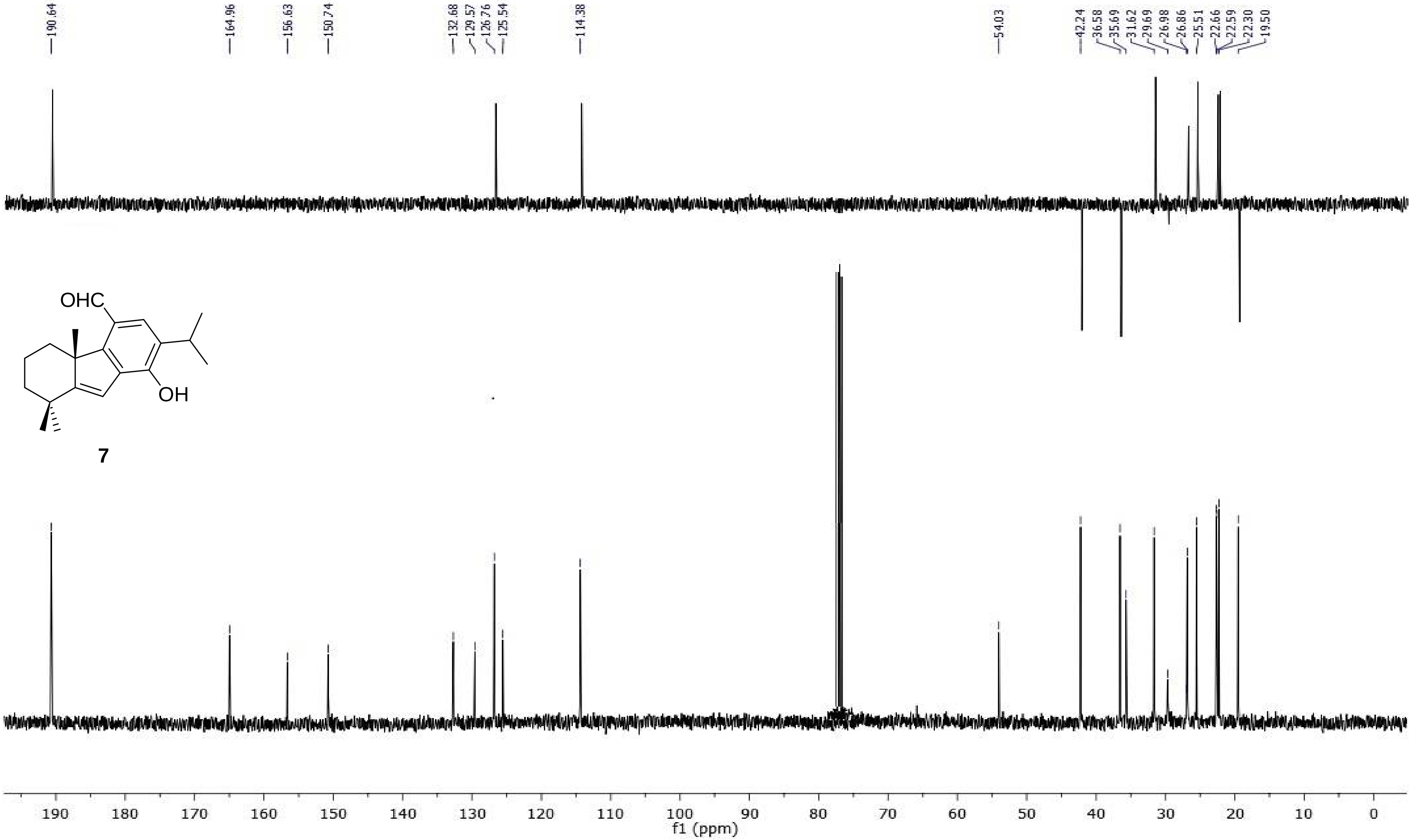
190.64
164.96
156.63
150.74
132.68
129.57
126.76
125.54
114.38
54.03
42.24
36.58
35.69
31.62
29.69
26.98
26.86
25.51
22.66
22.59
22.30
19.50
OHC
OH
7
190 180 170 160 150 140 130 120 110 100 90 80 70 60 50 40 30 20 10 0
f1 (ppm)

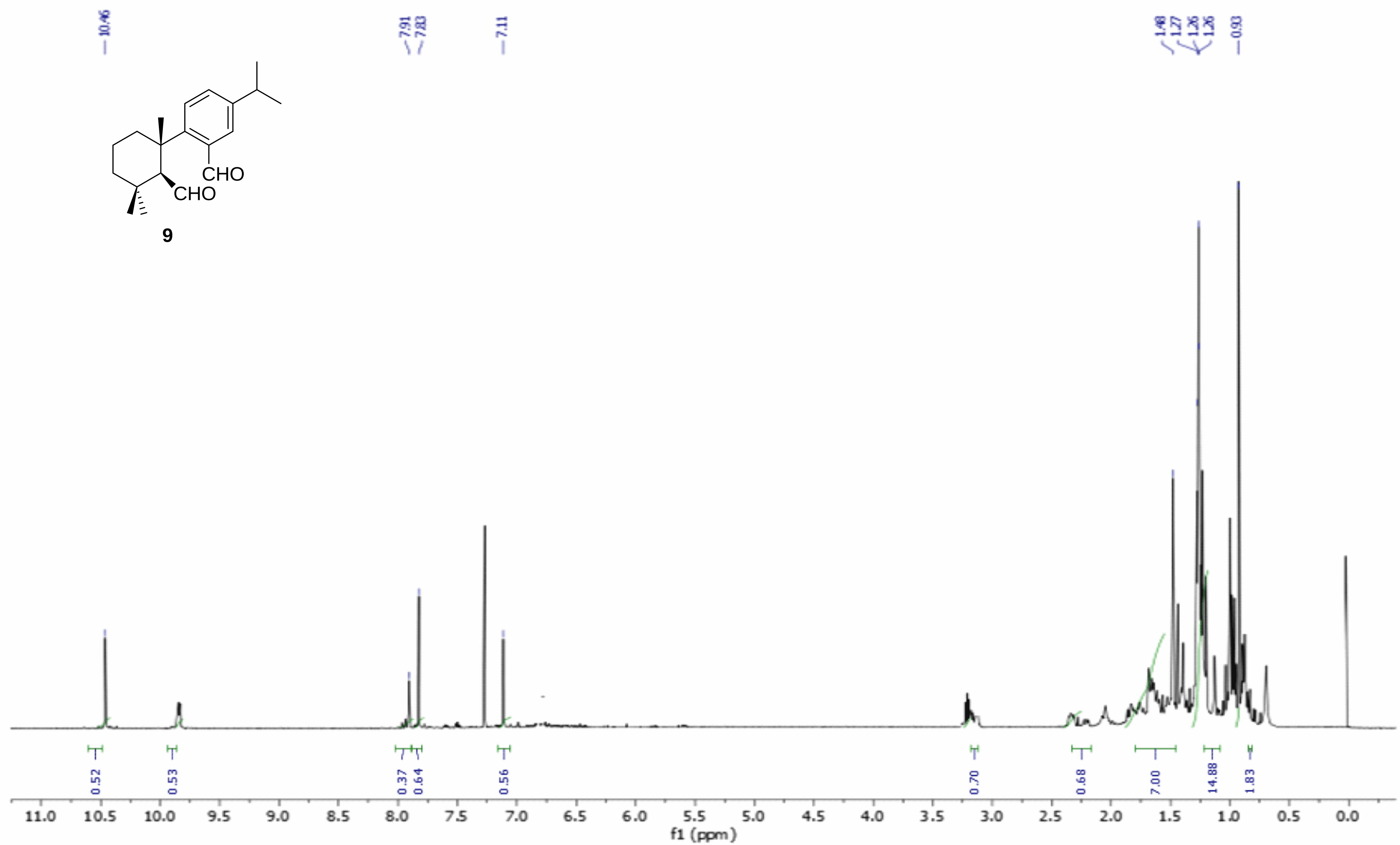
CHO
CHO
9
10.46
7.91
7.83
7.11
1.48
1.27
1.26
1.26
0.93
0.52
0.53
0.37
0.64
0.56
0.70
0.68
7.00
14.88
1.83
11.0 10.5 10.0 9.5 9.0 8.5 8.0 7.5 7.0 6.5 6.0 5.5 5.0 4.5 4.0 3.5 3.0 2.5 2.0 1.5 1.0 0.5 0.0
f1 (ppm)

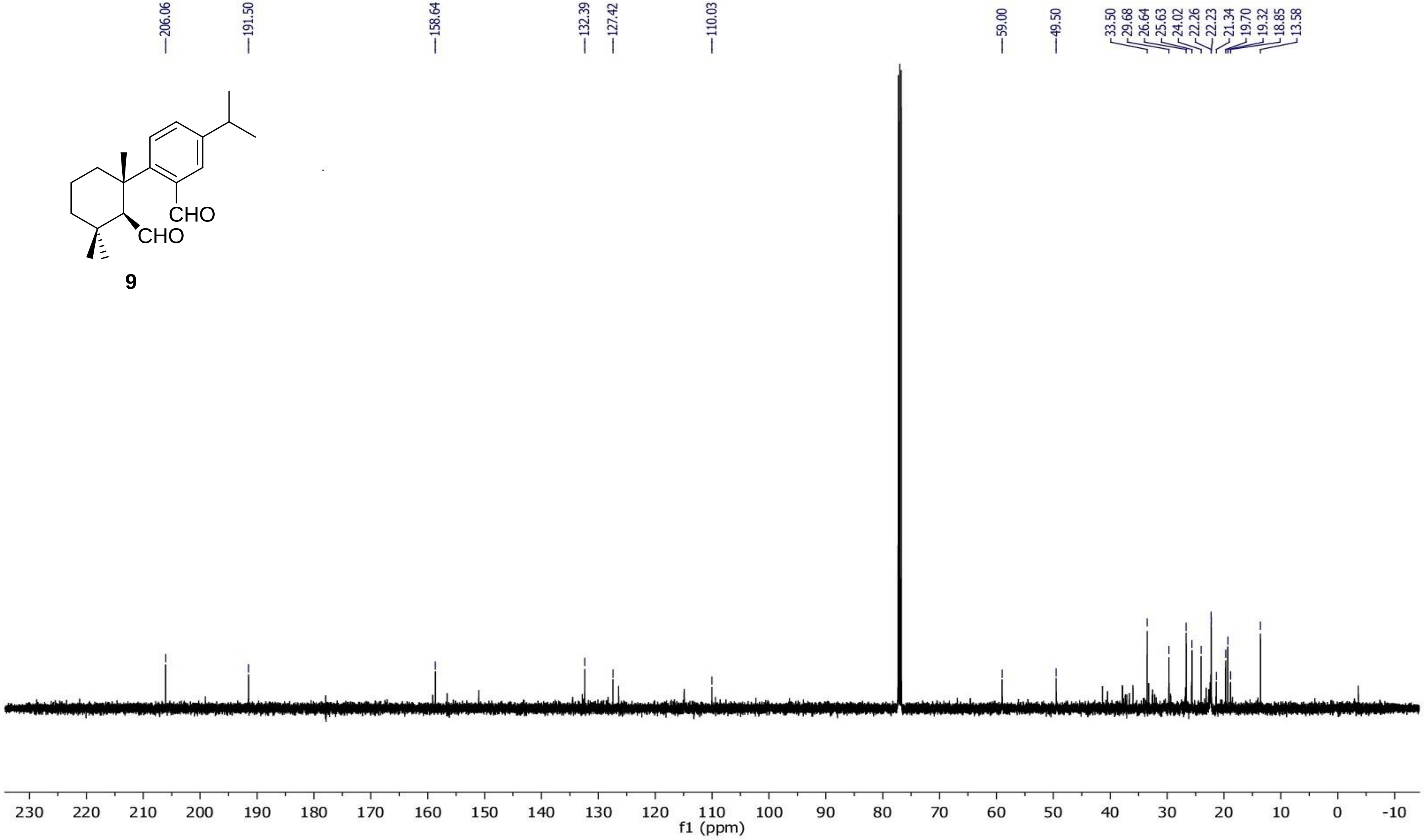
CHO
CHO
9
206.06
191.50
158.64
132.39
127.42
110.03
59.00
49.50
33.50
29.68
26.64
25.63
24.02
22.26
22.23
21.34
19.70
19.32
18.85
13.58
230 220 210 200 190 180 170 160 150 140 130 120 110 100 90 80 70 60 50 40 30 20 10 0 -10
f1 (ppm)

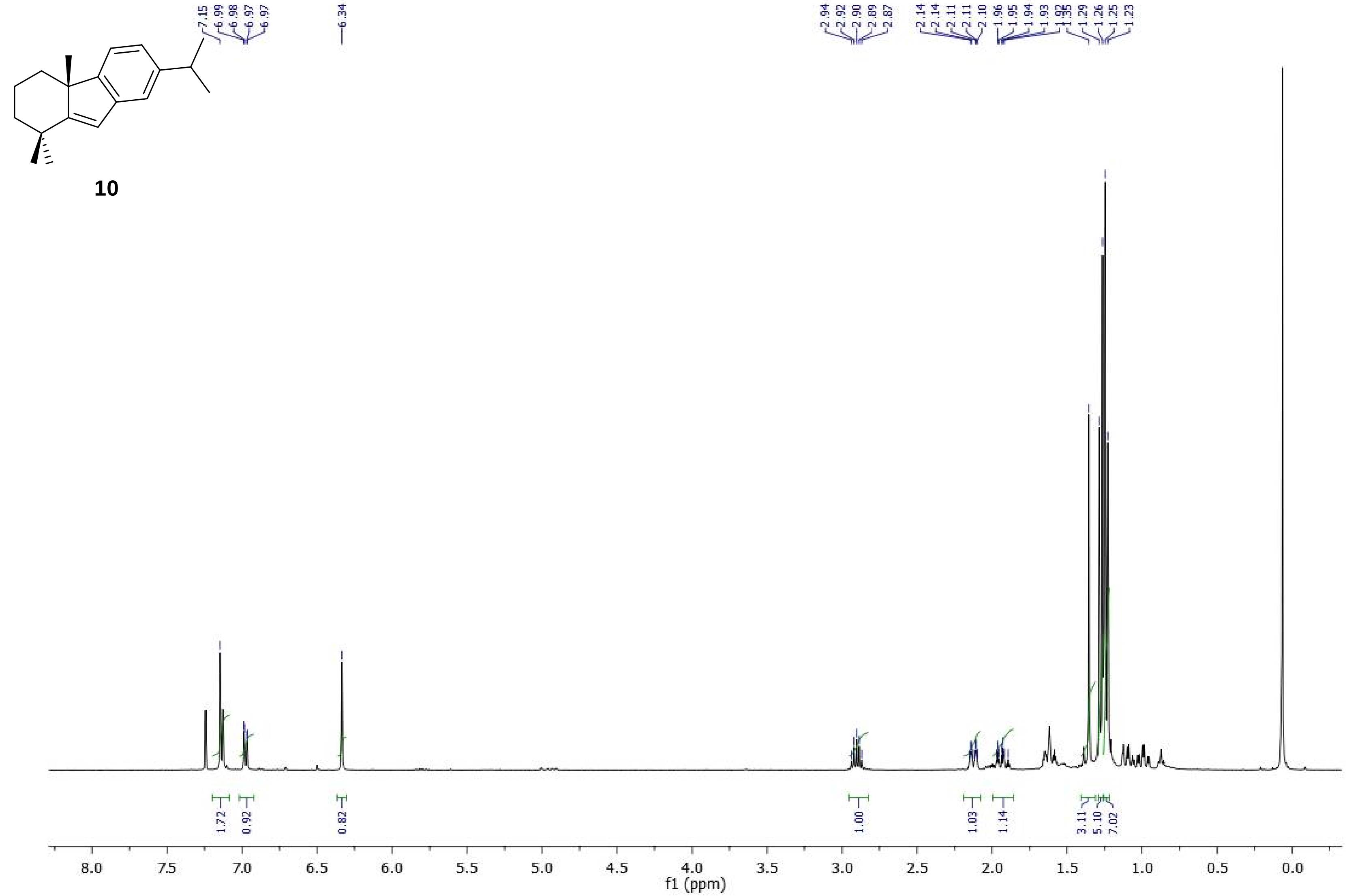

10

7.15
6.99
6.98
6.97
6.97
6.34
2.94
2.92
2.90
2.89
2.87
2.14
2.14
2.11
2.11
2.10
1.96
1.95
1.94
1.93
1.92
1.35
1.29
1.26
1.25
1.23
1.72
0.92
0.82
1.00
1.03
1.14
3.11
5.10
7.02
8.0
7.5
7.0
6.5
6.0
5.5
5.0
4.5
4.0
3.5
3.0
2.5
2.0
1.5
1.0
0.5
0.0
f1 (ppm)

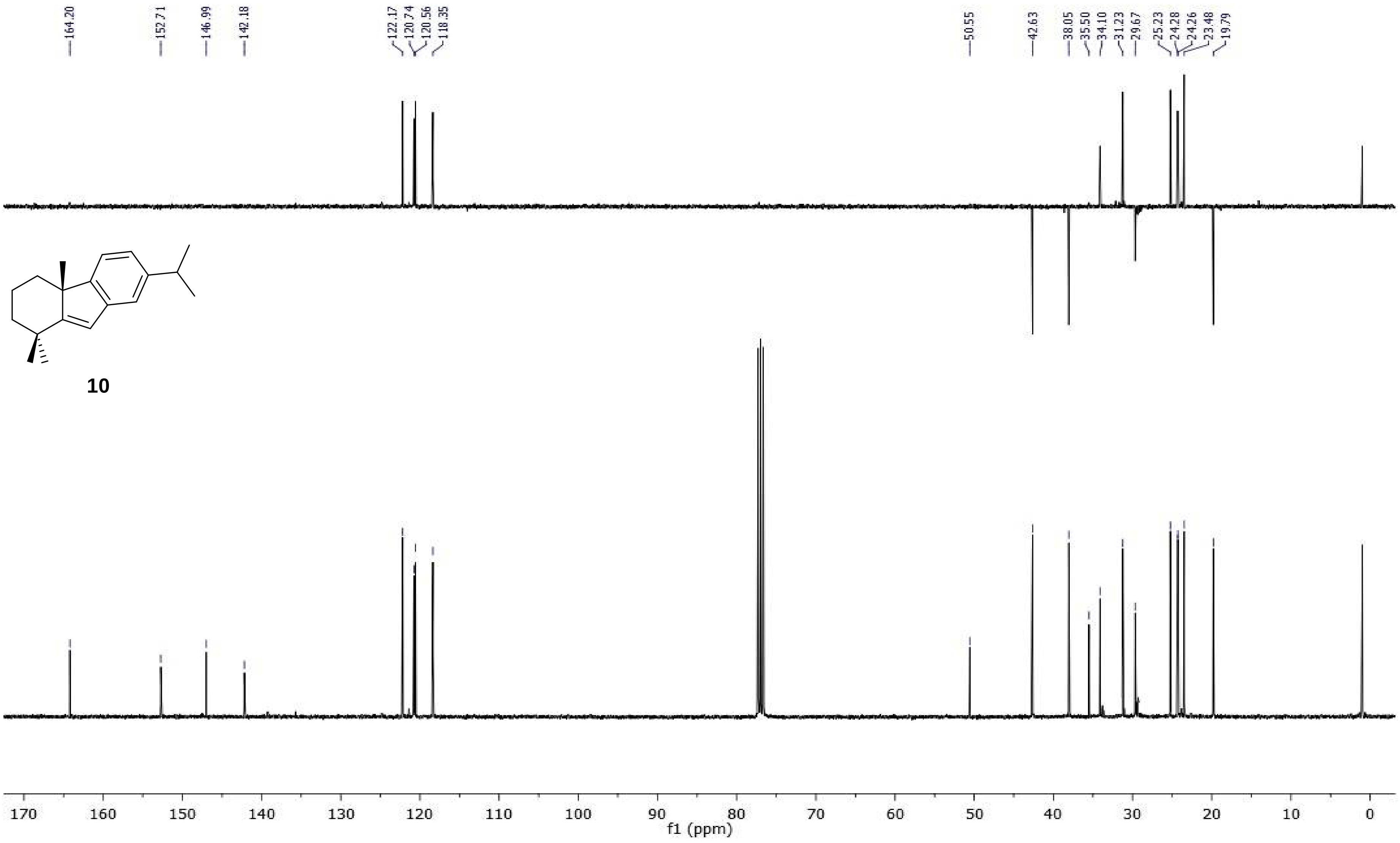
164.20
152.71
146.99
142.18
122.17
120.74
120.56
118.35
50.55
42.63
38.05
35.50
34.10
31.23
29.67
25.23
24.28
24.26
23.48
19.79
10
170
160
150
140
130
120
110
100
90
80
70
60
50
40
30
20
10
0
f1 (ppm)

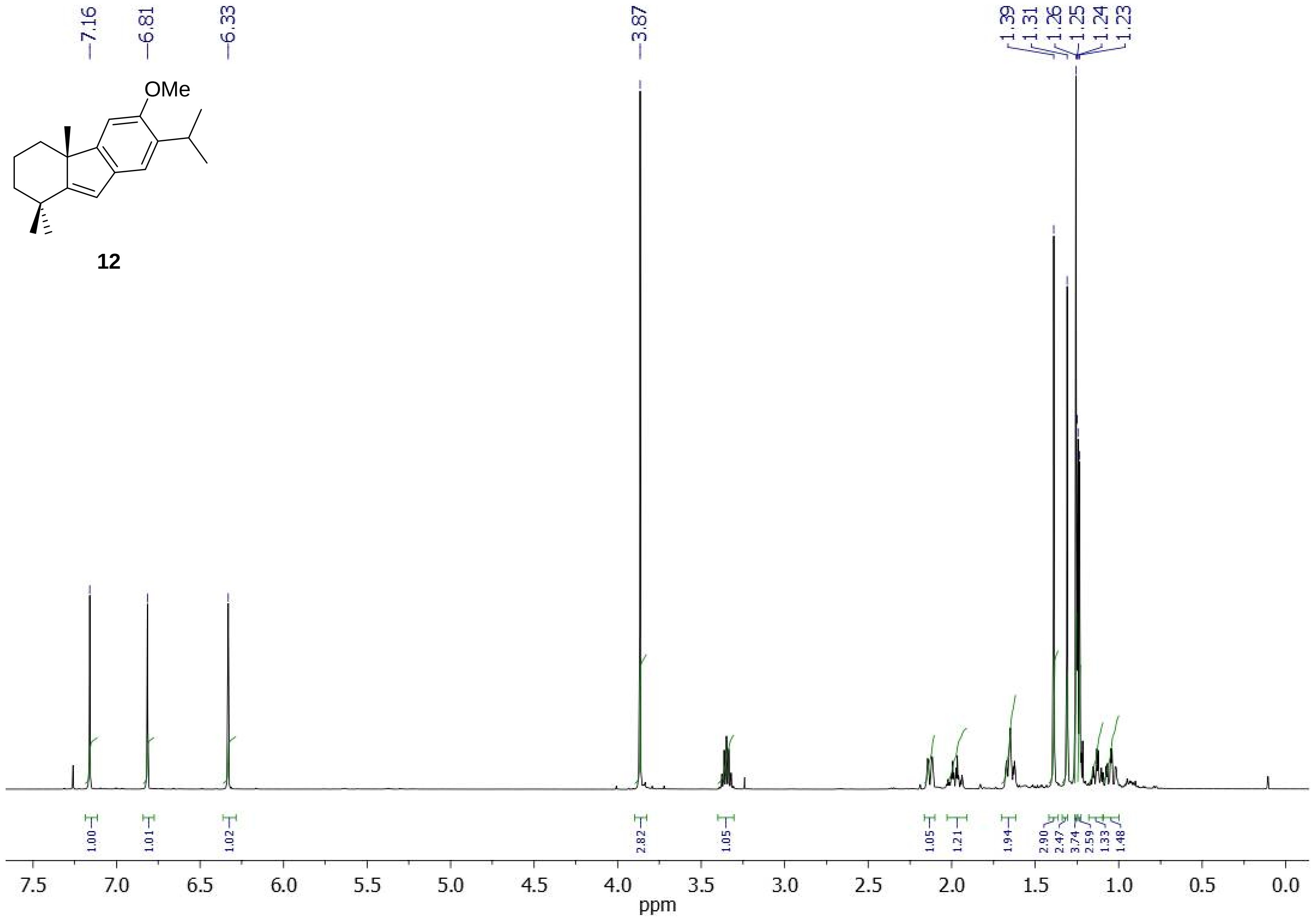
OMe
12
7.16
6.81
6.33
3.87
1.39
1.31
1.26
1.25
1.24
1.23
1.00
1.01
1.02
2.82
1.05
1.05
1.21
1.94
2.90
2.47
3.74
2.59
1.33
1.48
7.5 7.0 6.5 6.0 5.5 5.0 4.5 4.0 3.5 3.0 2.5 2.0 1.5 1.0 0.5 0.0
ppm

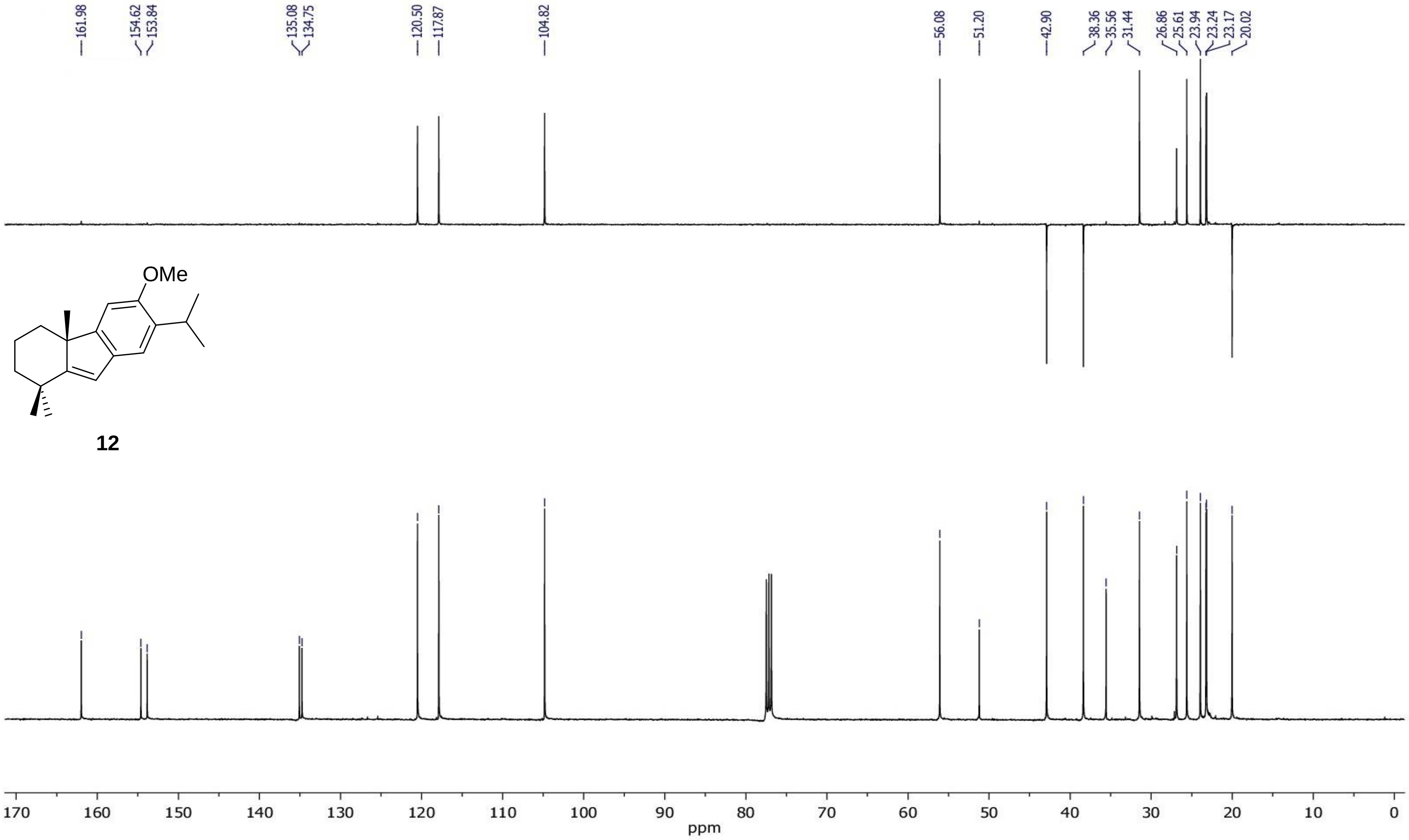
161.98
154.62
153.84
135.08
134.75
120.50
117.87
104.82
56.08
51.20
42.90
38.36
35.56
31.44
26.86
25.61
23.94
23.24
23.17
20.02
OMe
12
170
160
150
140
130
120
110
100
90
80
70
60
50
40
30
20
10
0
ppm

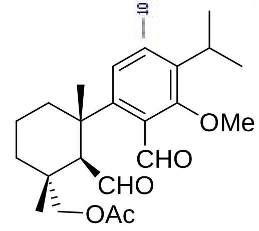
OMe
CHO
CHO
OAc

14

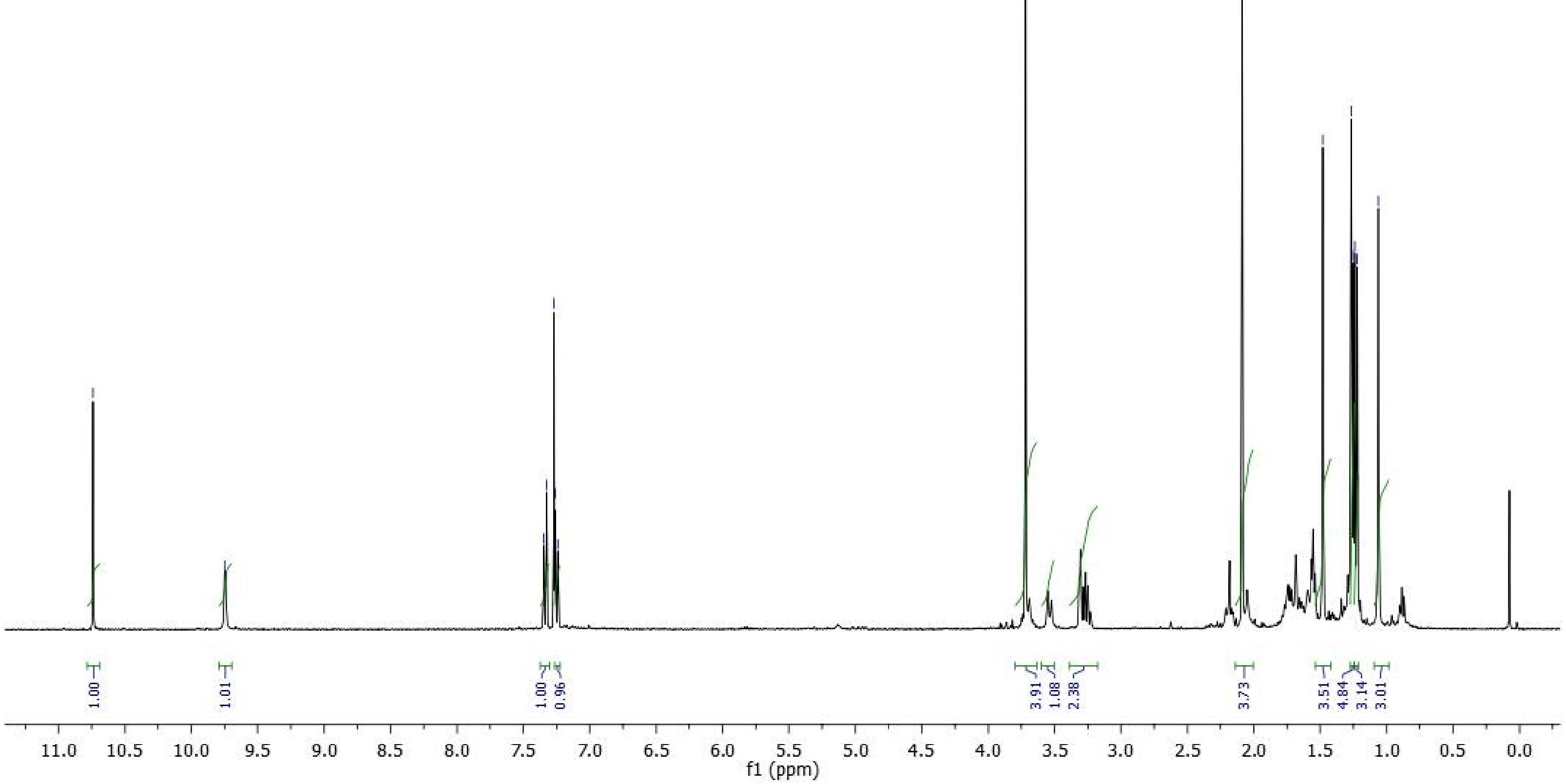
10.74
9.75
7.35
7.33
7.27
7.26
7.24
3.72
2.09
1.48
1.27
1.25
1.24
1.22
1.06
1.00
1.01
1.00
0.96
3.91
1.08
2.38
3.73
3.51
4.84
3.14
3.01
11.0 10.5 10.0 9.5 9.0 8.5 8.0 7.5 7.0 6.5 6.0 5.5 5.0 4.5 4.0 3.5 3.0 2.5 2.0 1.5 1.0 0.5 0.0
f1 (ppm)

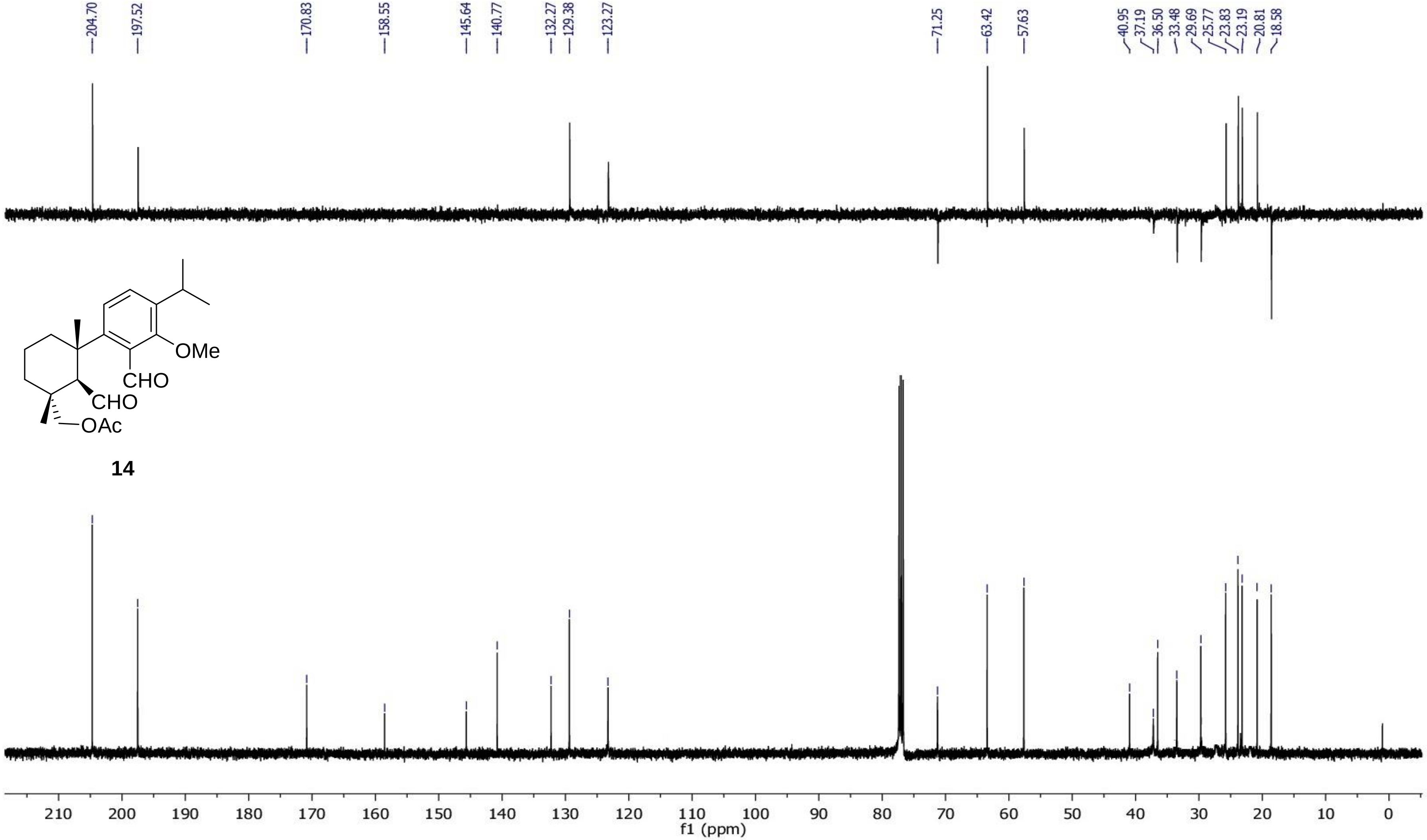
204.70
197.52
170.83
158.55
145.64
140.77
132.27
129.38
123.27
71.25
63.42
57.63
40.95
37.19
36.50
33.48
29.69
25.77
23.83
23.19
20.81
18.58
OMe
CHO
CHO
OAc
14
210 200 190 180 170 160 150 140 130 120 110 100 90 80 70 60 50 40 30 20 10 0
f1 (ppm)

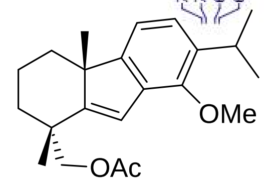
OMe
OAc

15

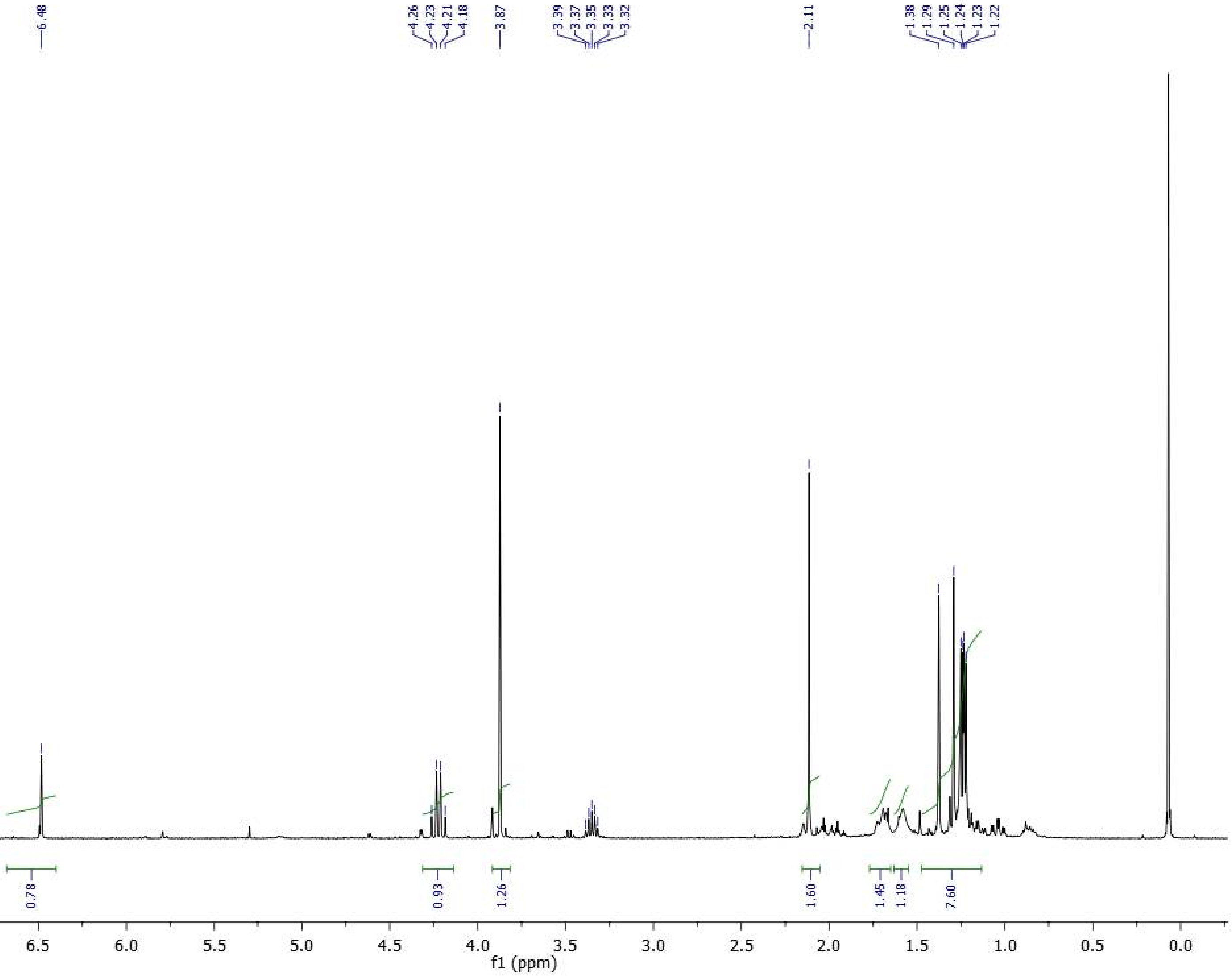
7.04
7.03
6.98
6.96
6.48
4.26
4.23
4.21
4.18
3.87
3.39
3.37
3.35
3.33
3.32
2.11
1.38
1.29
1.25
1.24
1.23
1.22
1.00
0.78
0.93
1.26
1.60
1.45
1.18
7.60
7.5
7.0
6.5
6.0
5.5
5.0
4.5
4.0
3.5
3.0
2.5
2.0
1.5
1.0
0.5
0.0
f1 (ppm)

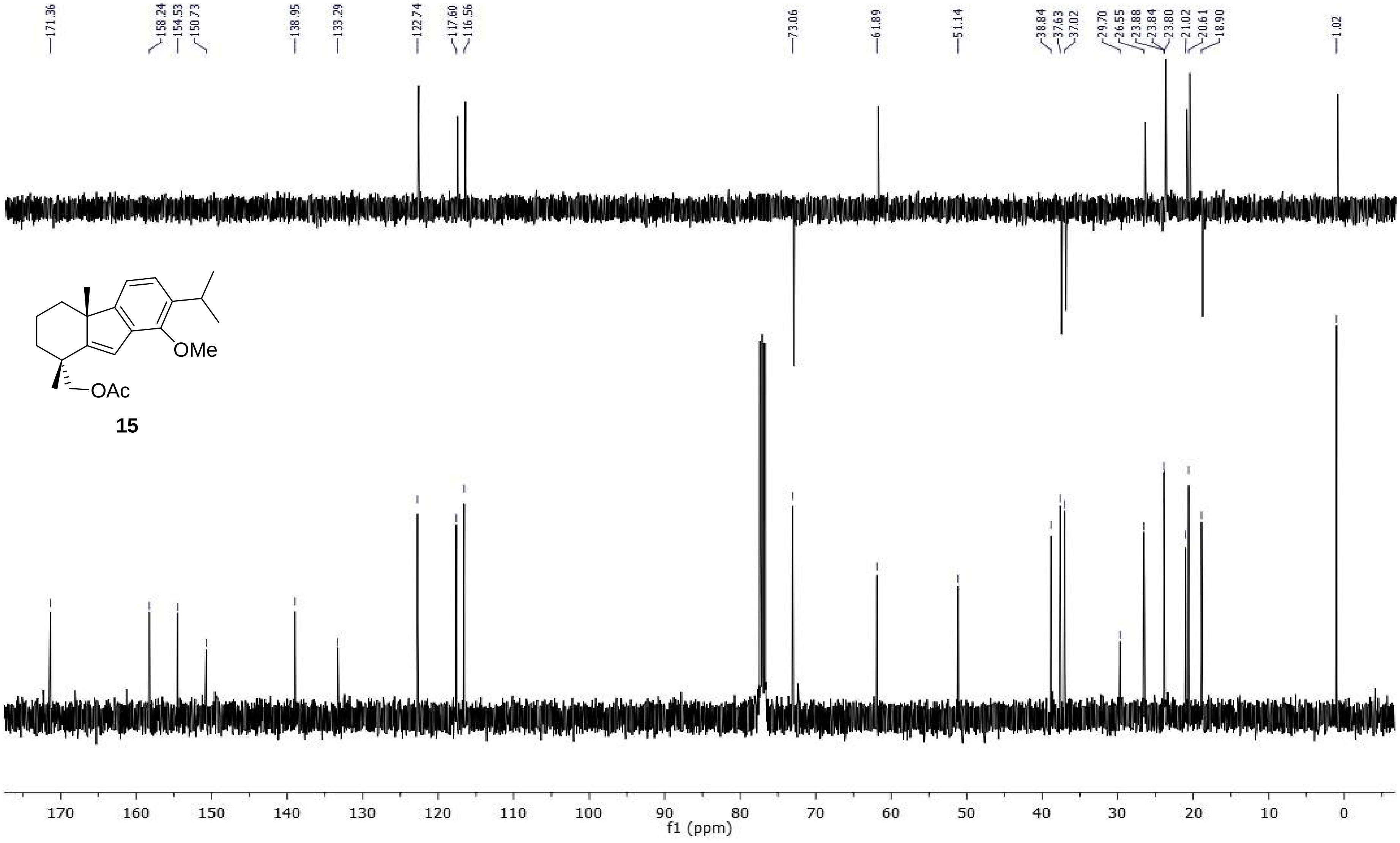

171.36
158.24
154.53
150.73
138.95
133.29
122.74
117.60
116.56
73.06
61.89
51.14
38.84
37.63
37.02
29.70
26.55
23.88
23.84
23.80
21.02
20.61
18.90
1.02
OMe
OAc
15
170 160 150 140 130 120 110 100 90 80 70 60 50 40 30 20 10 0
f1 (ppm)

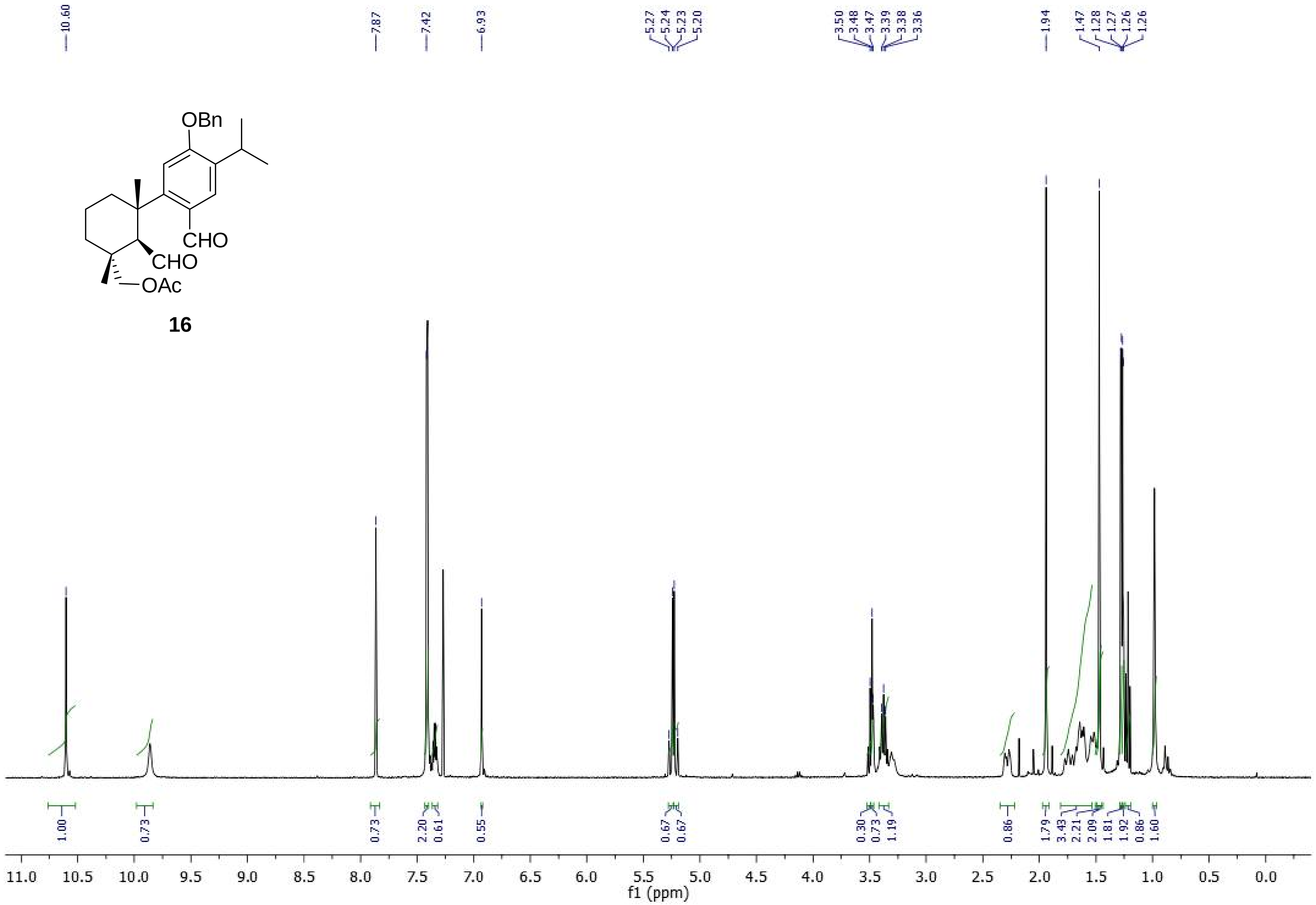

10.60
7.87
7.42
6.93
5.27
5.24
5.23
5.20
3.50
3.48
3.47
3.39
3.38
3.36
1.94
1.47
1.28
1.27
1.26
1.26
1.00
0.73
0.73
2.20
0.61
0.55
0.67
0.67
0.30
0.73
1.19
0.86
1.79
3.43
2.21
2.09
1.81
1.92
0.86
1.60
11.0
10.5
10.0
9.5
9.0
8.5
8.0
7.5
7.0
6.5
6.0
5.5
5.0
4.5
4.0
3.5
3.0
2.5
2.0
1.5
1.0
0.5
0.0
f1 (ppm)

16

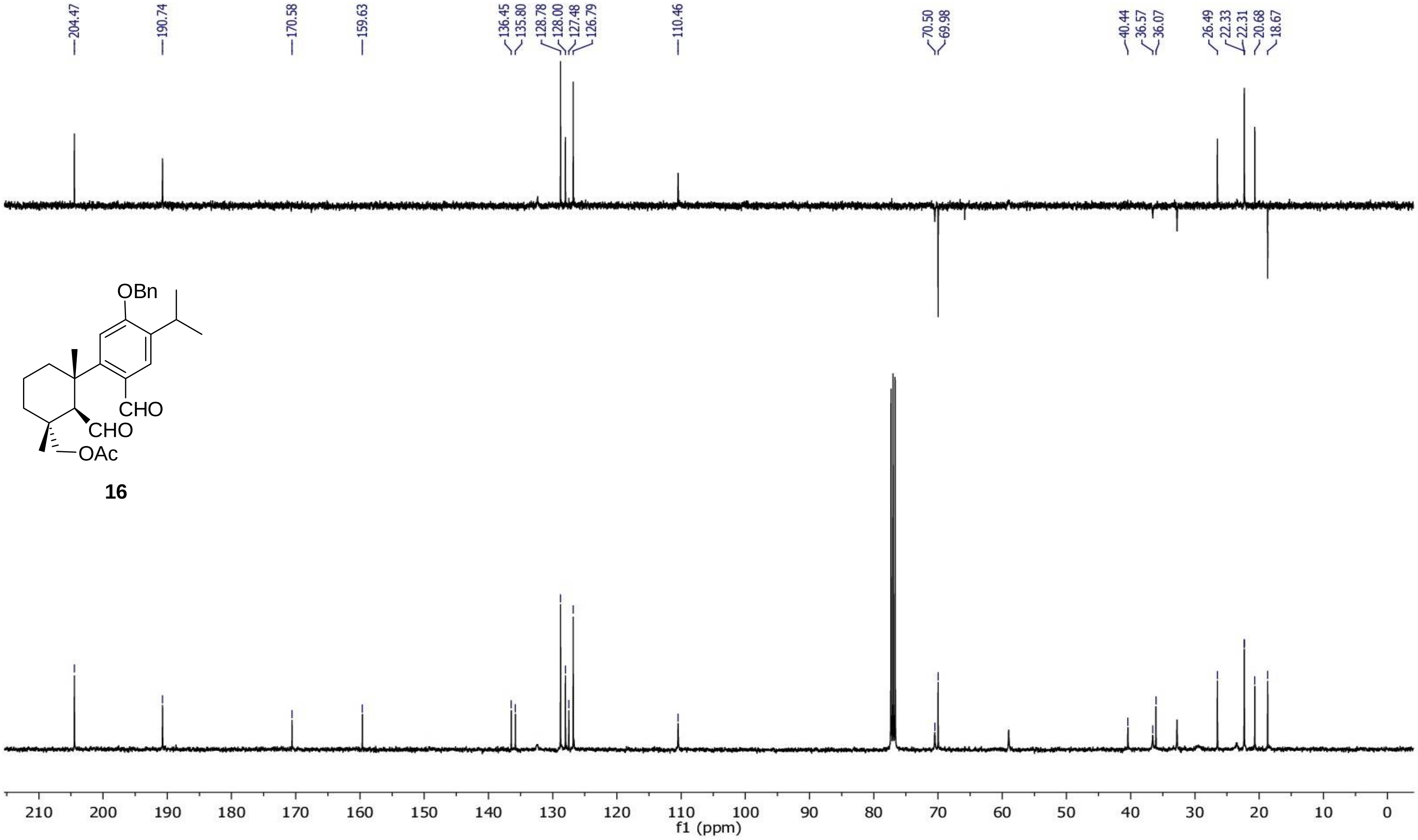
204.47
190.74
170.58
159.63
136.45
135.80
128.78
128.00
127.48
126.79
110.46
70.50
69.98
40.44
36.57
36.07
26.49
22.33
22.31
20.68
18.67
210 200 190 180 170 160 150 140 130 120 110 100 90 80 70 60 50 40 30 20 10 0
f1 (ppm)
OBn
CHO
CHO
OAc

16

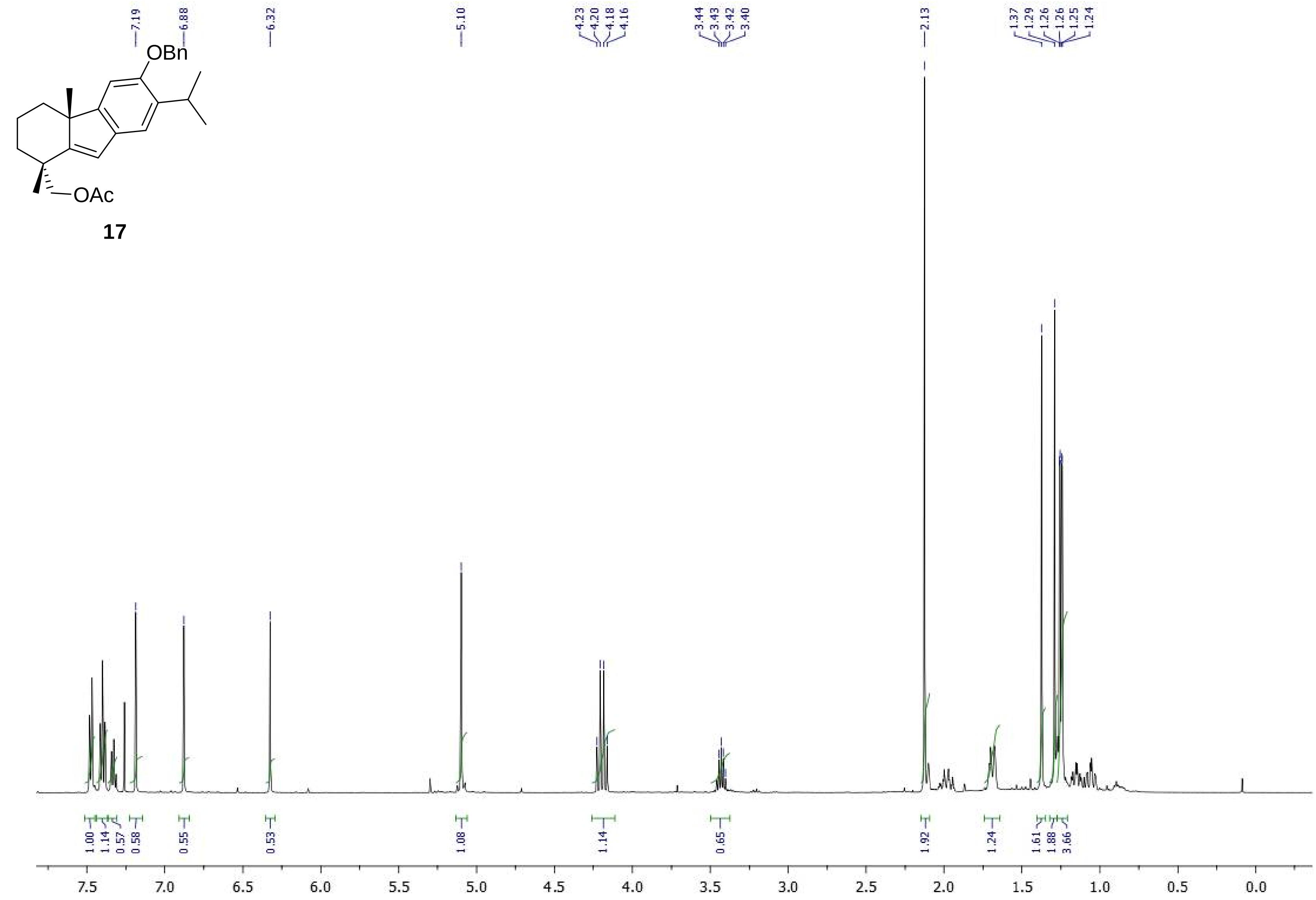
OBn
OAc
17
7.19
6.88
6.32
5.10
4.23
4.20
4.18
4.16
3.44
3.43
3.42
3.40
2.13
1.37
1.29
1.26
1.26
1.25
1.24
1.00
1.14
0.57
0.58
0.55
0.53
1.08
1.14
0.65
1.92
1.24
1.61
1.88
3.66
7.5
7.0
6.5
6.0
5.5
5.0
4.5
4.0
3.5
3.0
2.5
2.0
1.5
1.0
0.5
0.0

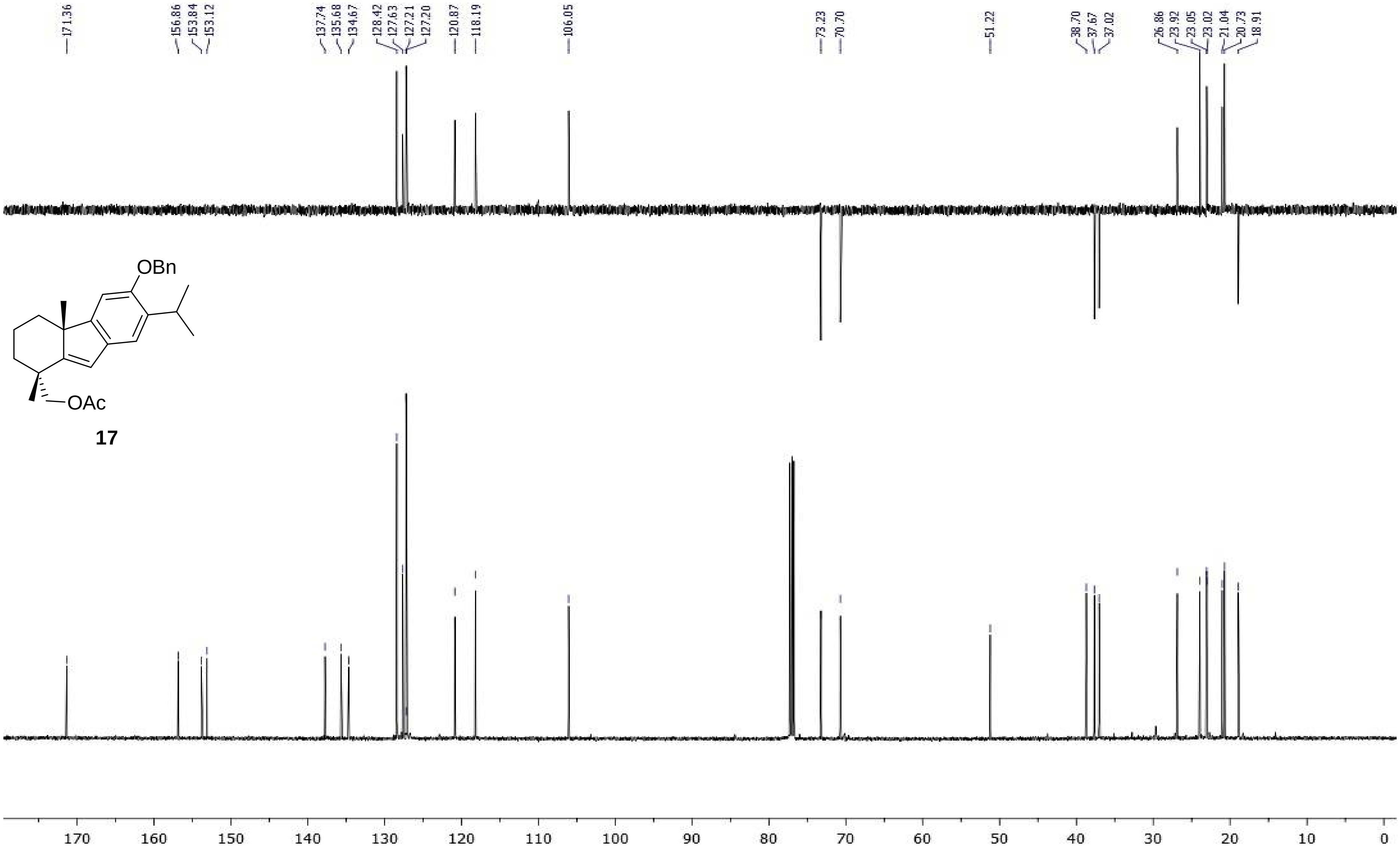

171.36
156.86
153.84
153.12
137.74
135.68
134.67
128.42
127.63
127.21
127.20
120.87
118.19
106.05
73.23
70.70
51.22
38.70
37.67
37.02
26.86
23.92
23.05
23.02
21.04
20.73
18.91
OBn
OAc
17
170 160 150 140 130 120 110 100 90 80 70 60 50 40 30 20 10 0

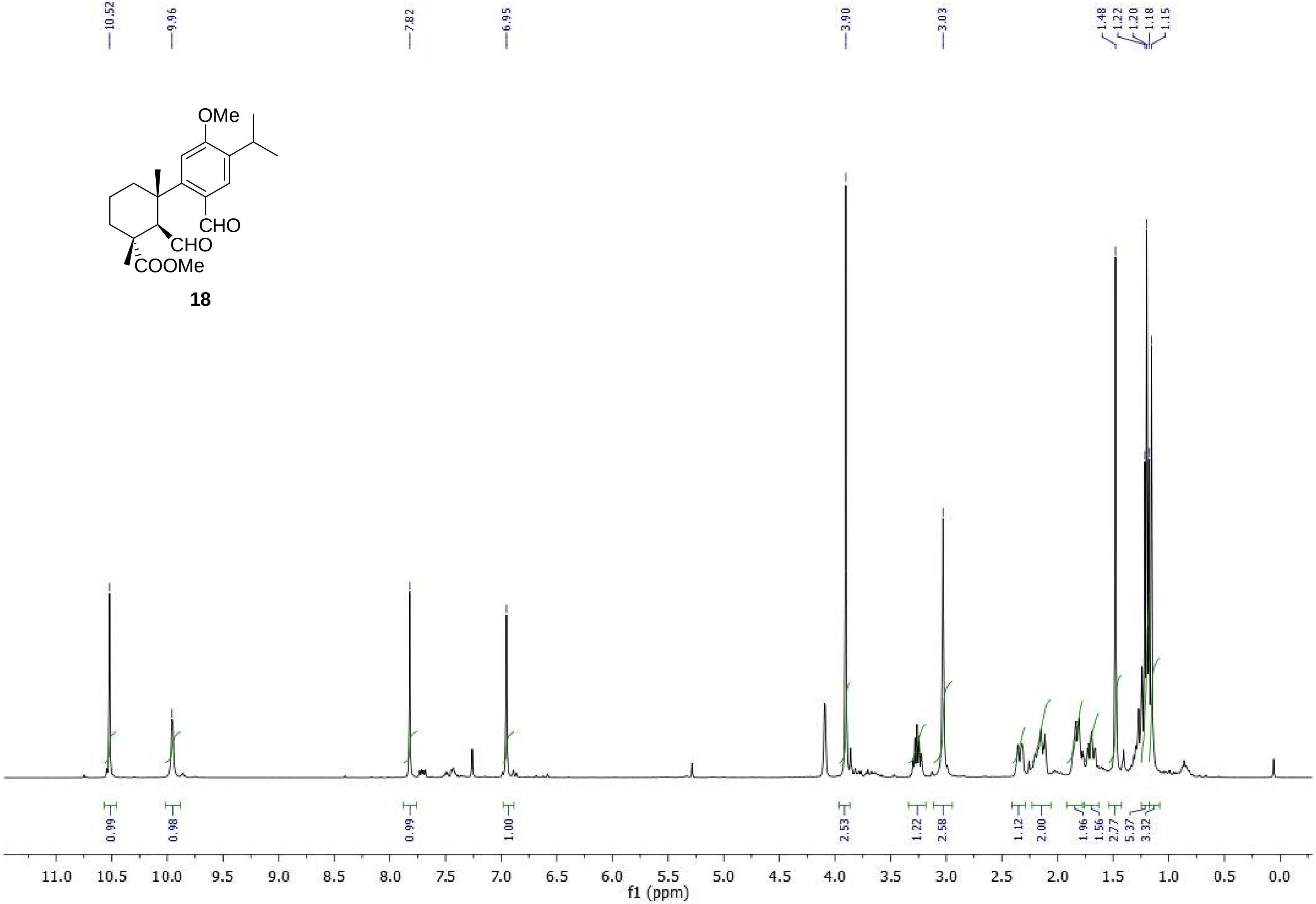
10.52
9.96
7.82
6.95
3.90
3.03
1.48
1.22
1.20
1.18
1.15
OMe
CHO
CHO
COOMe
18
0.99
0.98
0.99
1.00
2.53
1.22
2.58
1.12
2.00
1.96
1.56
2.77
5.37
3.32
11.0
10.5
10.0
9.5
9.0
8.5
8.0
7.5
7.0
6.5
6.0
5.5
5.0
4.5
4.0
3.5
3.0
2.5
2.0
1.5
1.0
0.5
0.0
f1 (ppm)

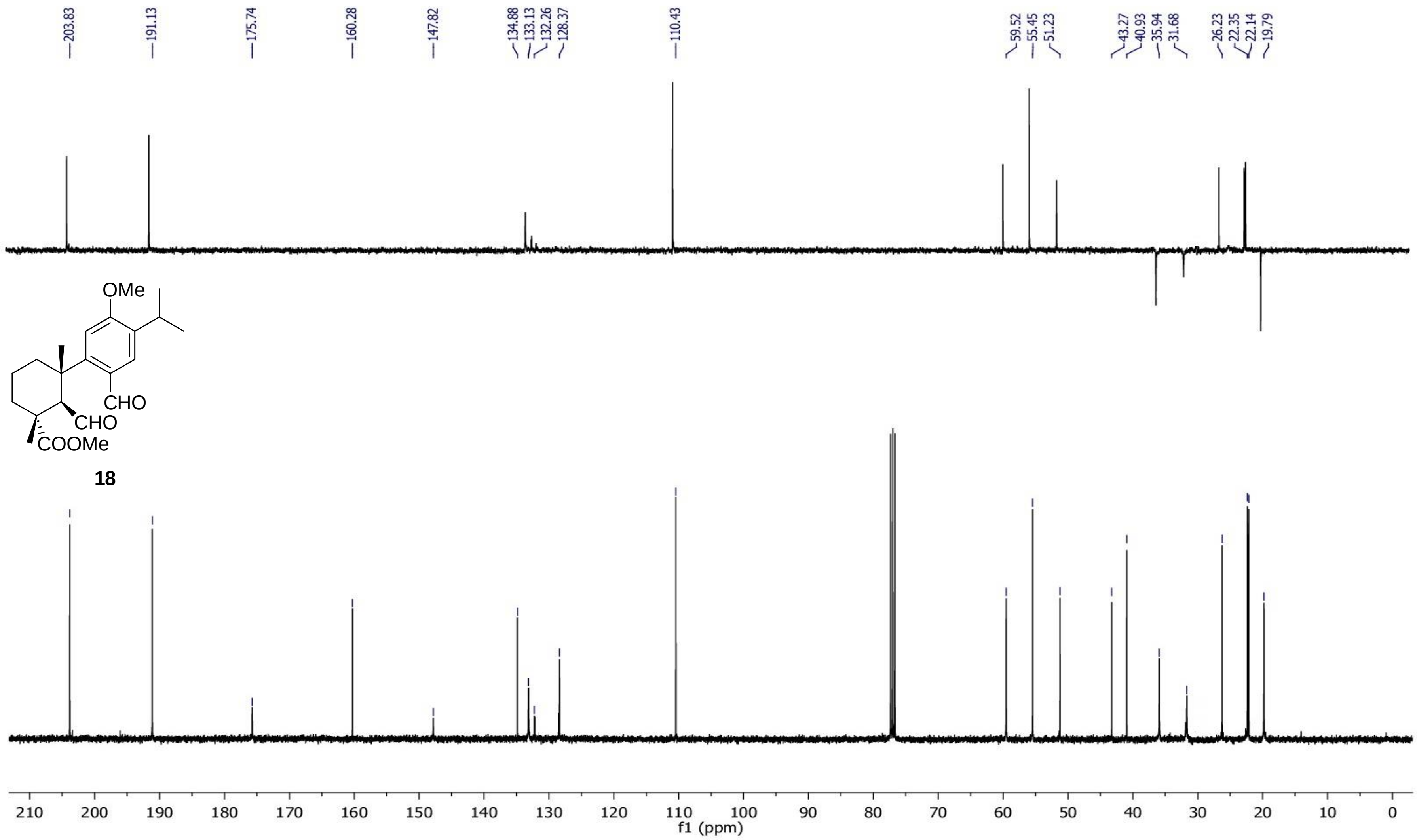
203.83
191.13
175.74
160.28
147.82
134.88
133.13
132.26
128.37
110.43
59.52
55.45
51.23
43.27
40.93
35.94
31.68
26.23
22.35
22.14
19.79
OMe
CHO
CHO
COOMe
18
210 200 190 180 170 160 150 140 130 120 110 100 90 80 70 60 50 40 30 20 10 0
f1 (ppm)

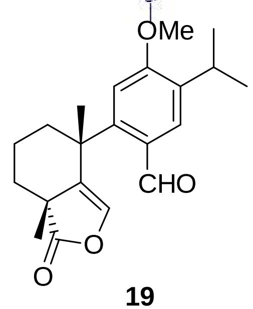
OMe
CHO
O
O
O

19

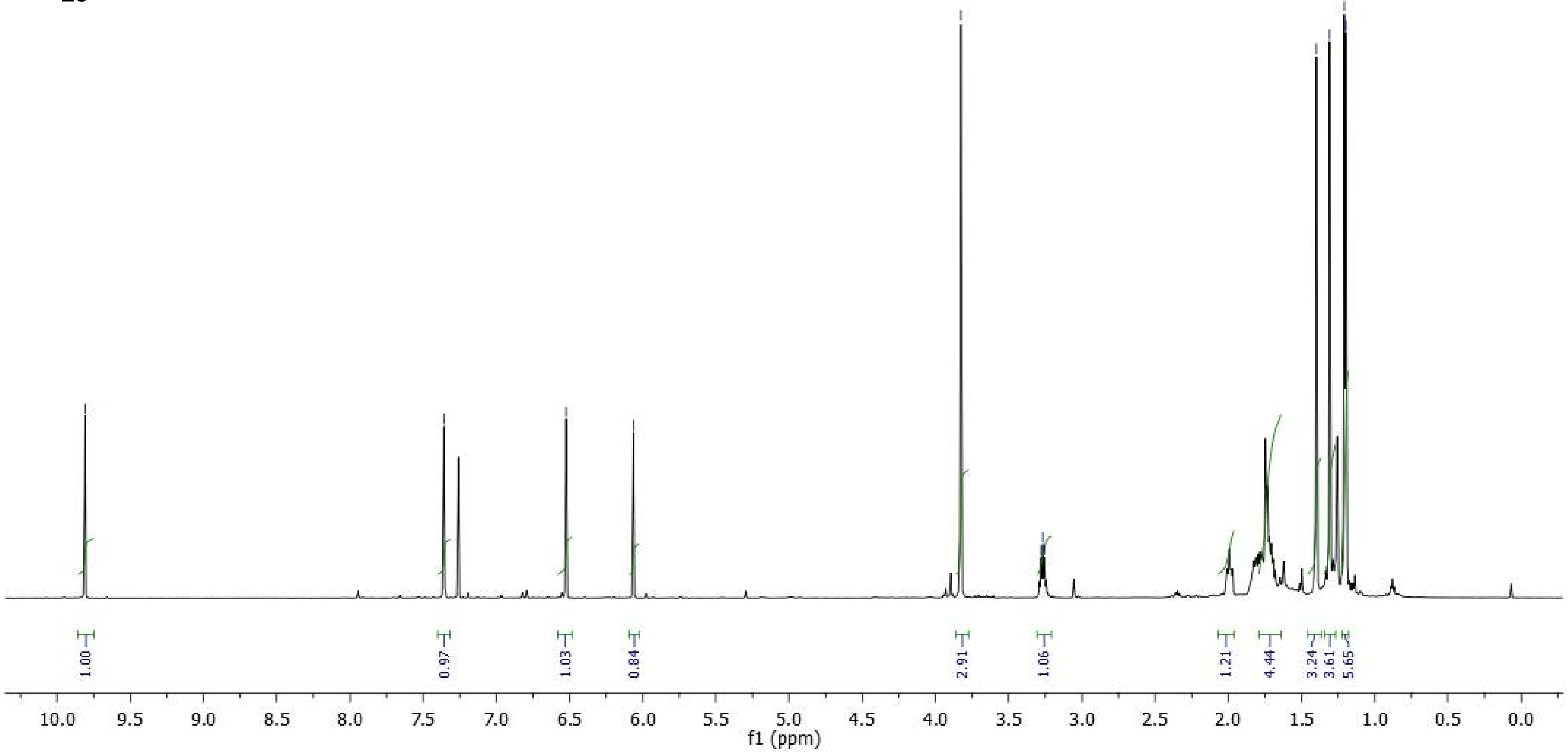
9.81
7.36
6.52
6.06
3.83
3.28
3.27
3.26
1.40
1.31
1.21
1.20
1.00
0.97
1.03
0.84
2.91
1.06
1.21
4.44
3.24
3.61
5.65
10.0 9.5 9.0 8.5 8.0 7.5 7.0 6.5 6.0 5.5 5.0 4.5 4.0 3.5 3.0 2.5 2.0 1.5 1.0 0.5 0.0
f1 (ppm)

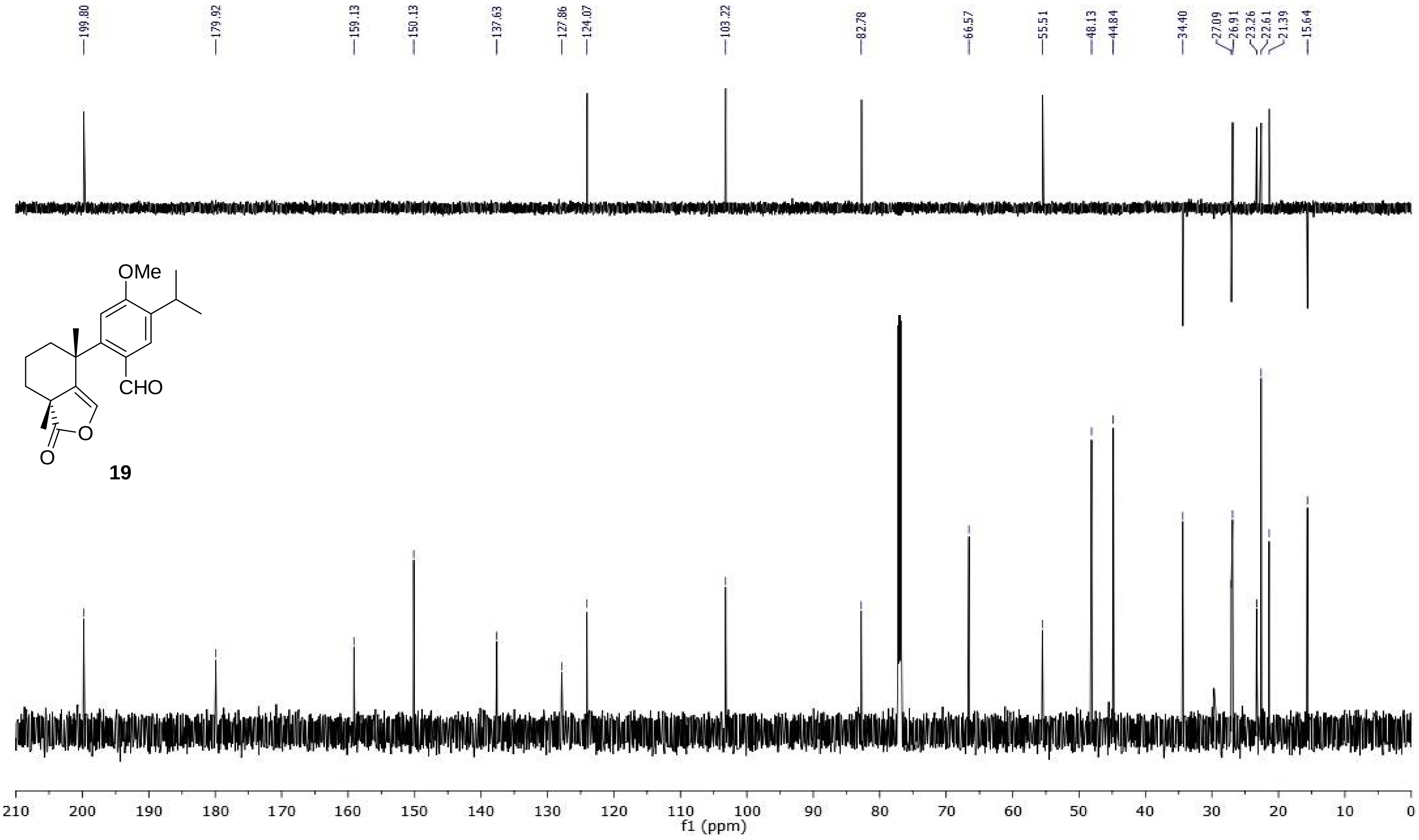

199.80
179.92
159.13
150.13
137.63
127.86
124.07
103.22
82.78
66.57
55.51
48.13
44.84
34.40
27.09
26.91
23.26
22.61
21.39
15.64
OMe
CHO
O
O
19
210 200 190 180 170 160 150 140 130 120 110 100 90 80 70 60 50 40 30 20 10 0
f1 (ppm)

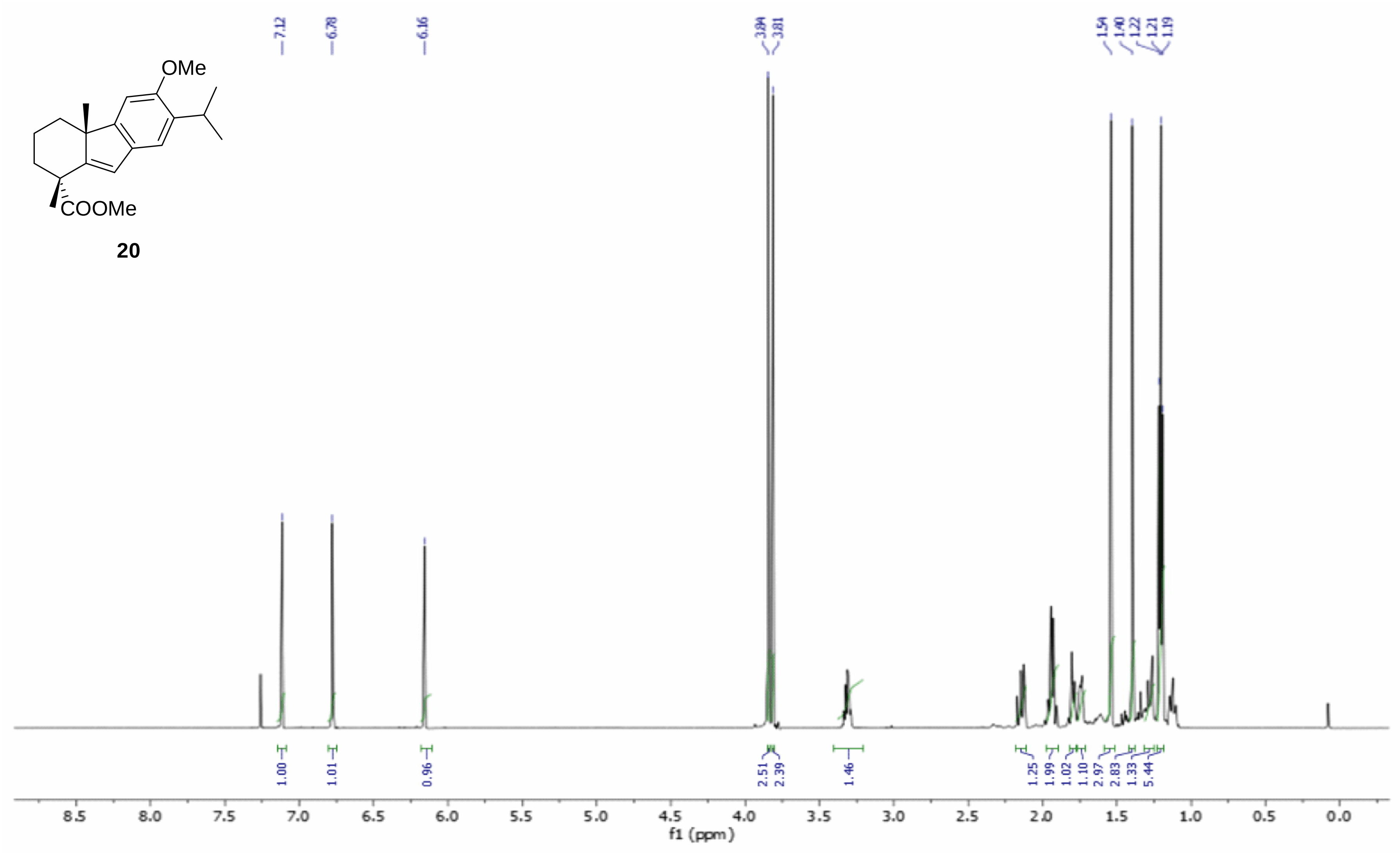
OMe
COOMe
20
7.12
6.78
6.16
3.84
3.81
1.54
1.40
1.22
1.21
1.19
1.00
1.01
0.96
2.51
2.39
1.46
1.25
1.99
1.02
1.10
2.97
2.83
1.33
5.44
8.5
8.0
7.5
7.0
6.5
6.0
5.5
5.0
4.5
4.0
3.5
3.0
2.5
2.0
1.5
1.0
0.5
0.0
f1 (ppm)

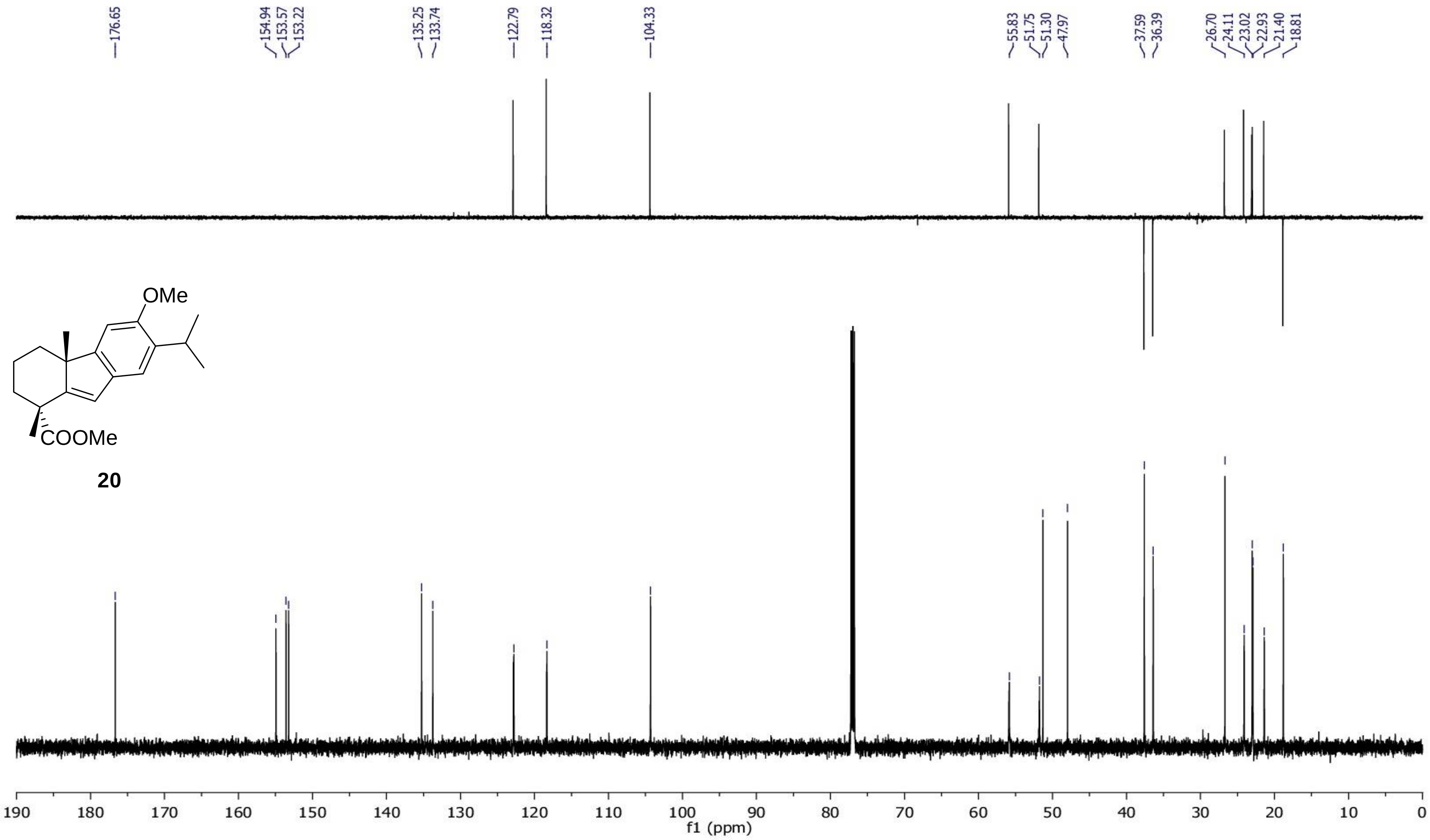
176.65
154.94
153.57
153.22
135.25
133.74
122.79
118.32
104.33
55.83
51.75
51.30
47.97
37.59
36.39
26.70
24.11
23.02
22.93
21.40
18.81
OMe
COOMe
190
180
170
160
150
140
130
120
110
100
90
80
70
60
50
40
30
20
10
0
f1 (ppm)

20

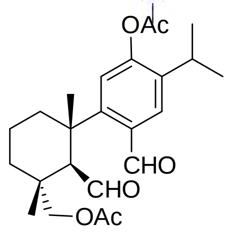
OAc
CHO
CHO
OAc

21

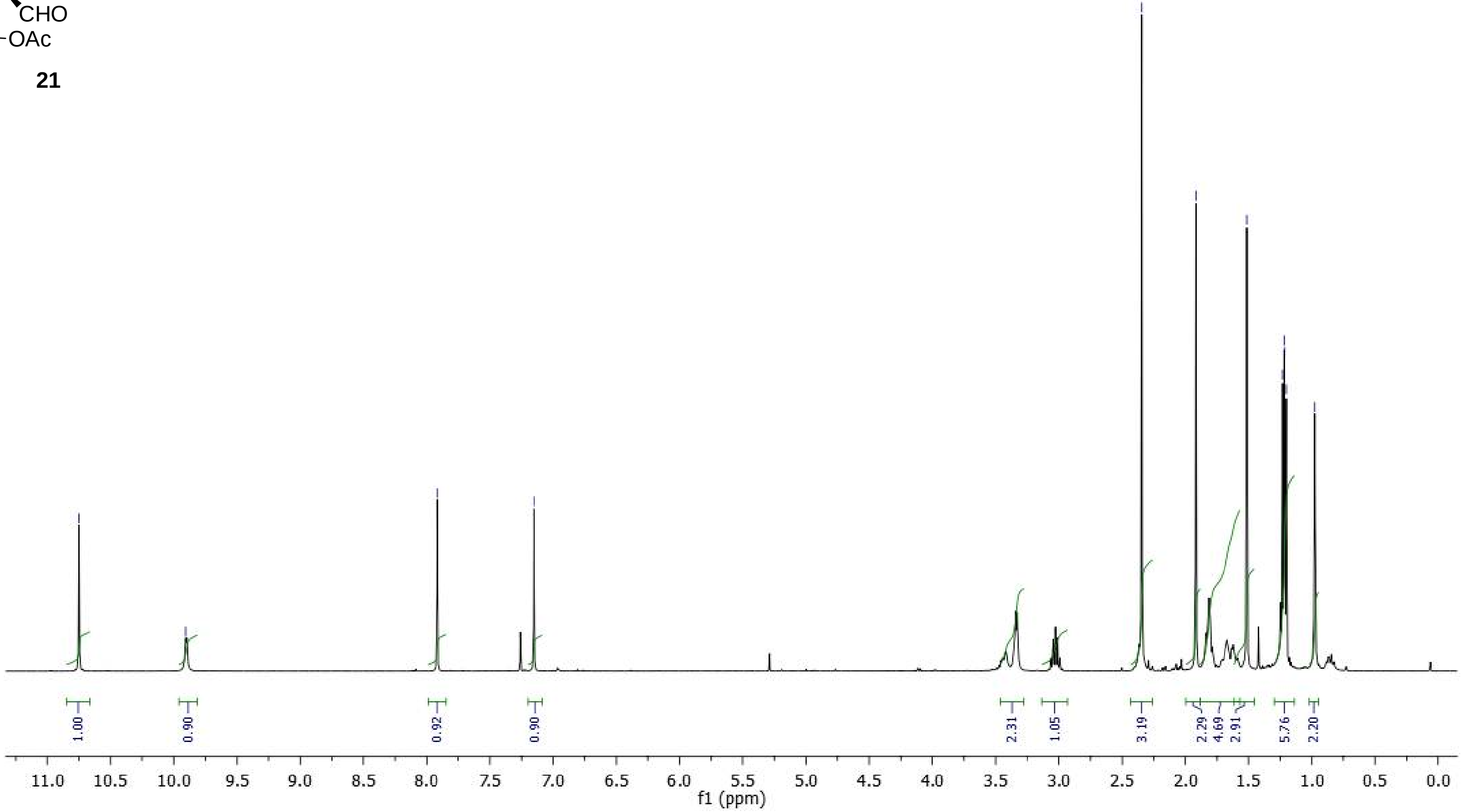
10.75
9.91
7.92
7.15
2.34
1.92
1.51
1.23
1.22
1.21
1.20
0.98
1.00
0.90
0.92
0.90
2.31
1.05
3.19
2.29
4.69
2.91
5.76
2.20
11.0 10.5 10.0 9.5 9.0 8.5 8.0 7.5 7.0 6.5 6.0 5.5 5.0 4.5 4.0 3.5 3.0 2.5 2.0 1.5 1.0 0.5 0.0
f1 (ppm)

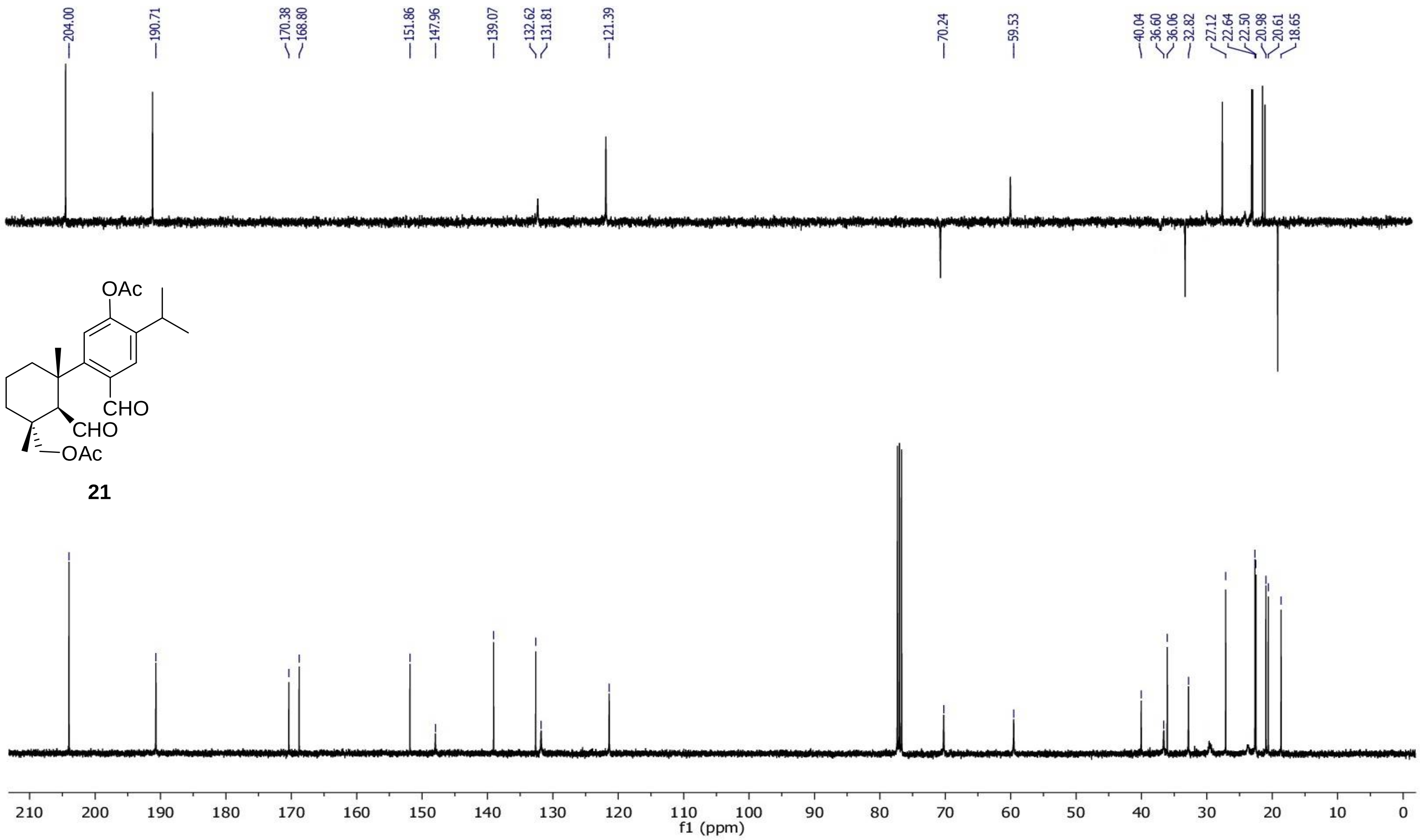
204.00
190.71
170.38
168.80
151.86
147.96
139.07
132.62
131.81
121.39
70.24
59.53
40.04
36.60
36.06
32.82
27.12
22.64
22.50
20.98
20.61
18.65
OAc
CHO
CHO
OAc
21
210 200 190 180 170 160 150 140 130 120 110 100 90 80 70 60 50 40 30 20 10 0
f1 (ppm)

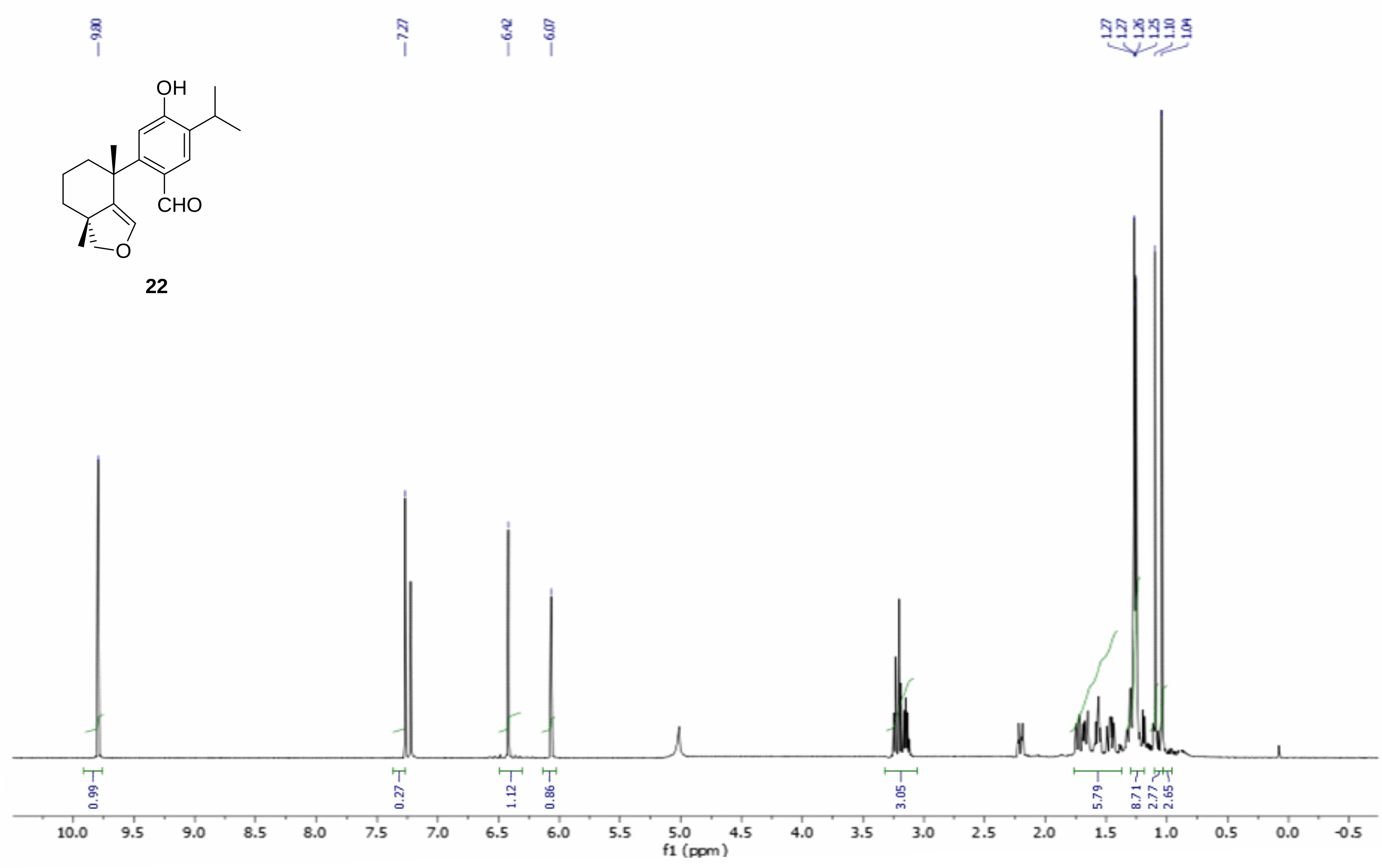
OH
CHO
O
22
9.80
7.27
6.42
6.07
1.27
1.27
1.26
1.25
1.10
1.04
0.99
0.27
1.12
0.86
3.05
5.79
8.71
2.77
2.65
10.0
9.5
9.0
8.5
8.0
7.5
7.0
6.5
6.0
5.5
5.0
4.5
4.0
3.5
3.0
2.5
2.0
1.5
1.0
0.5
0.0
-0.5
f1 (ppm)

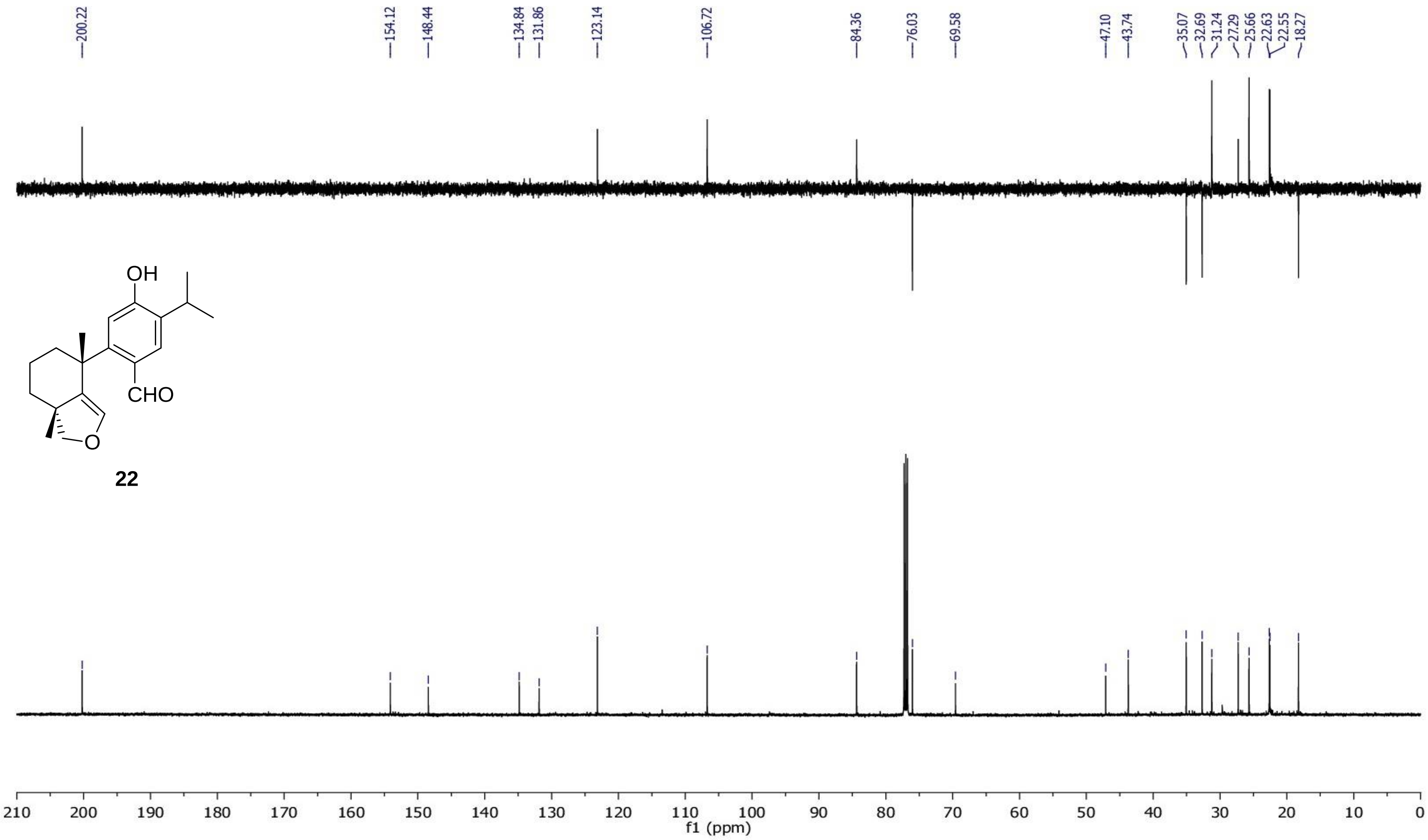
200.22
154.12
148.44
134.84
131.86
123.14
106.72
84.36
76.03
69.58
47.10
43.74
35.07
32.69
31.24
27.29
25.66
22.63
22.55
18.27
OH
CHO
O
22
210 200 190 180 170 160 150 140 130 120 110 100 90 80 70 60 50 40 30 20 10 0
f1 (ppm)

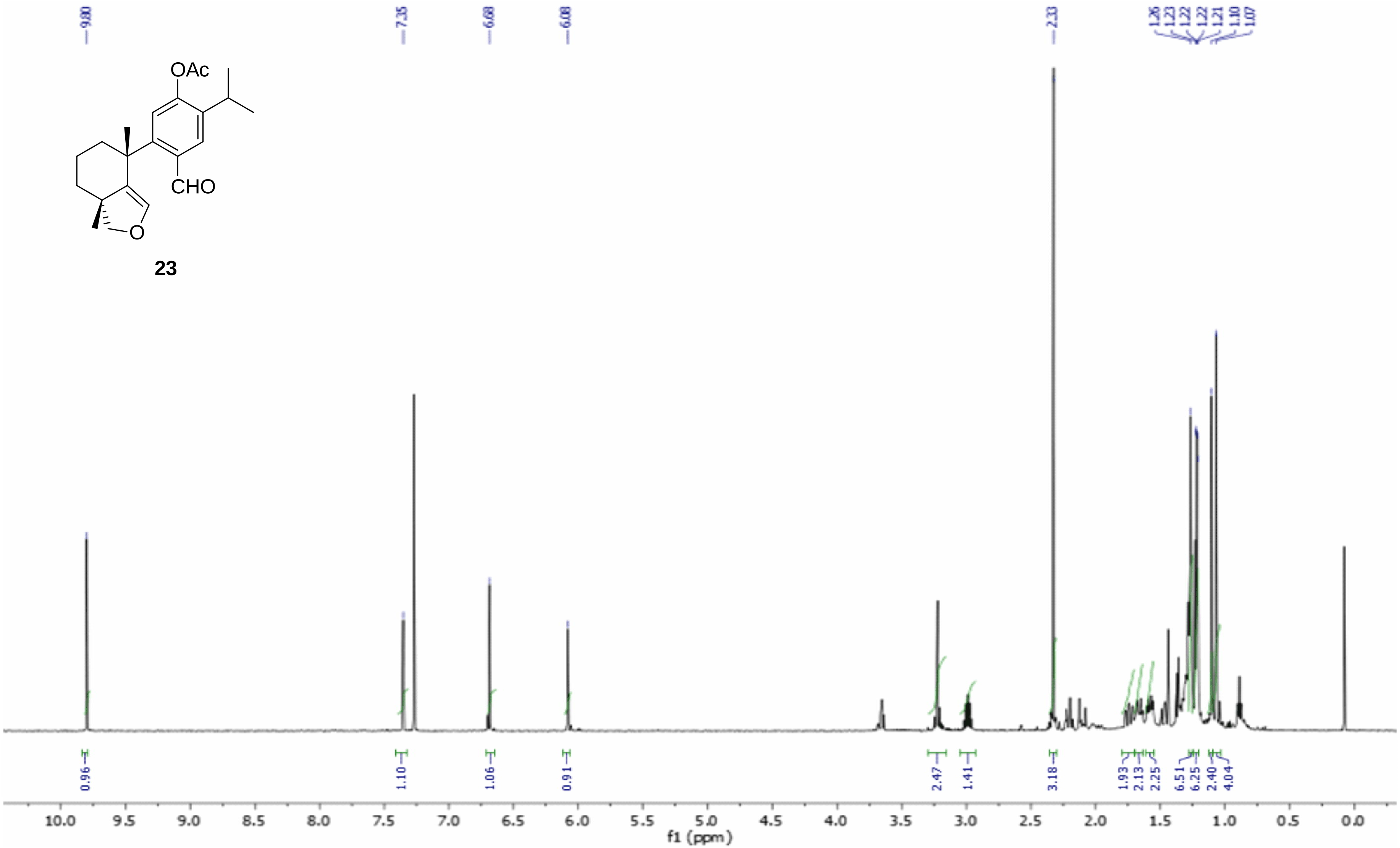

23

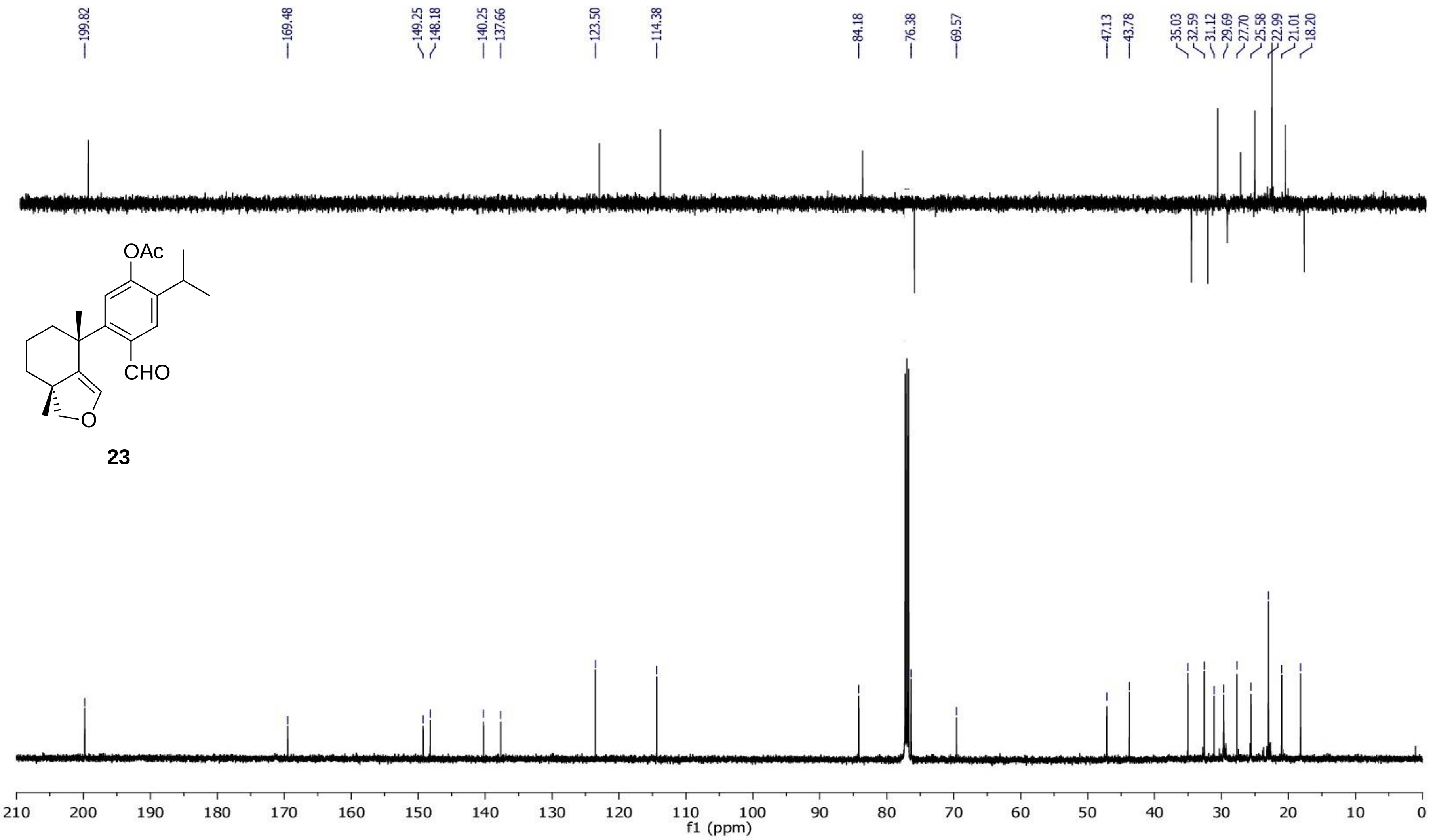
199.82
169.48
149.25
148.18
140.25
137.66
123.50
114.38
84.18
76.38
69.57
47.13
43.78
35.03
32.59
31.12
29.69
27.70
25.58
22.99
21.01
18.20
OAc
CHO
O
23
210 200 190 180 170 160 150 140 130 120 110 100 90 80 70 60 50 40 30 20 10 0
f1 (ppm)

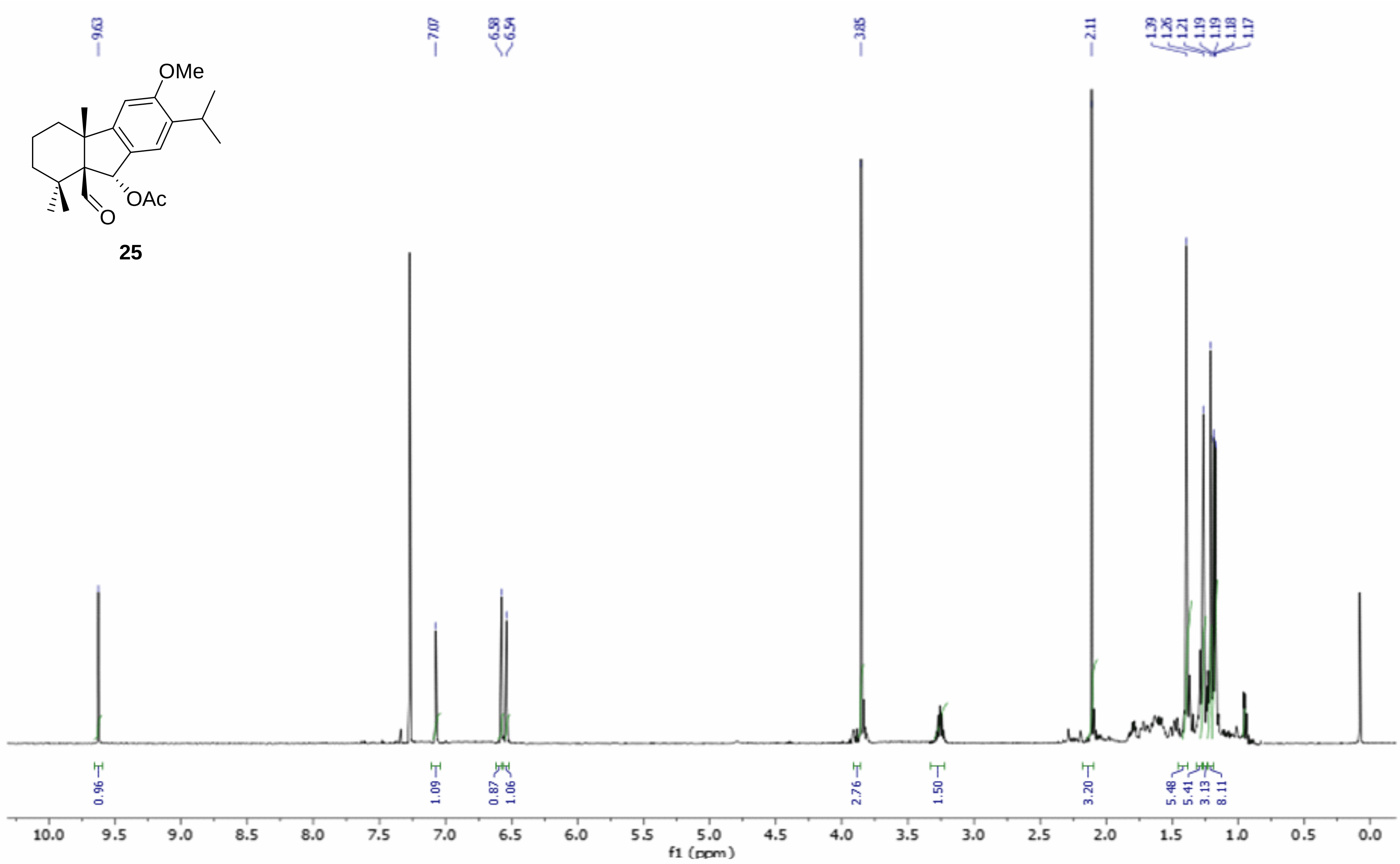
OMe
OAc
O
25
9.63
7.07
6.58
6.54
3.85
2.11
1.39
1.26
1.21
1.19
1.19
1.18
1.17
0.96
1.09
0.87
1.06
2.76
1.50
3.20
5.48
5.41
3.13
8.11
f1 (ppm)

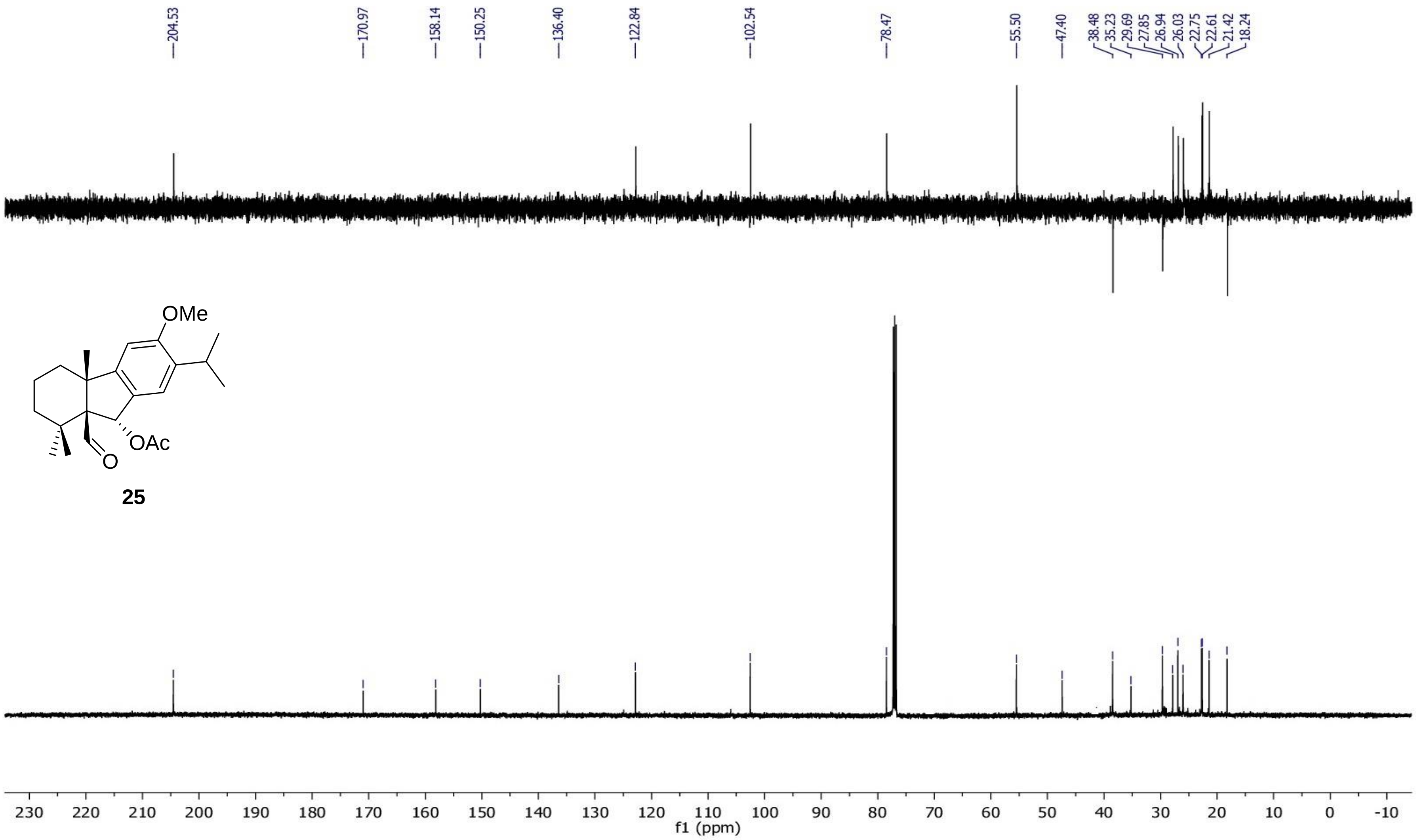
204.53
170.97
158.14
150.25
136.40
122.84
102.54
78.47
55.50
47.40
38.48
35.23
29.69
27.85
26.94
26.03
22.75
22.61
21.42
18.24
230 220 210 200 190 180 170 160 150 140 130 120 110 100 90 80 70 60 50 40 30 20 10 0 -10
f1 (ppm)
OMe
OAc
O

25

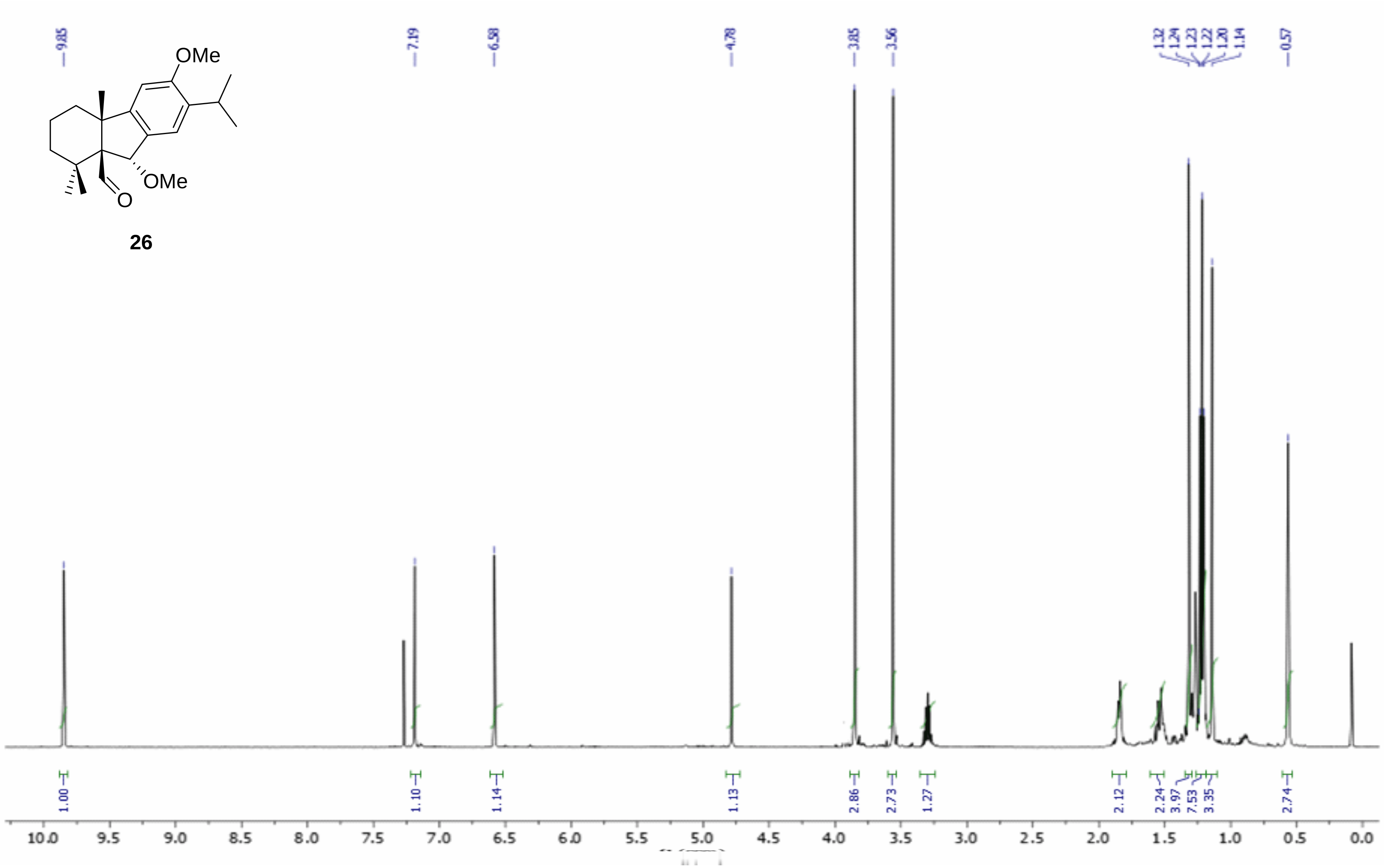
OMe
OMe
O
26
9.85
7.19
6.58
4.78
3.85
3.56
1.32
1.24
1.23
1.22
1.20
1.14
0.57
1.00
1.10
1.14
1.13
2.86
2.73
1.27
2.12
2.24
3.97
7.53
3.35
2.74
10.0
9.5
9.0
8.5
8.0
7.5
7.0
6.5
6.0
5.5
5.0
4.5
4.0
3.5
3.0
2.5
2.0
1.5
1.0
0.5
0.0

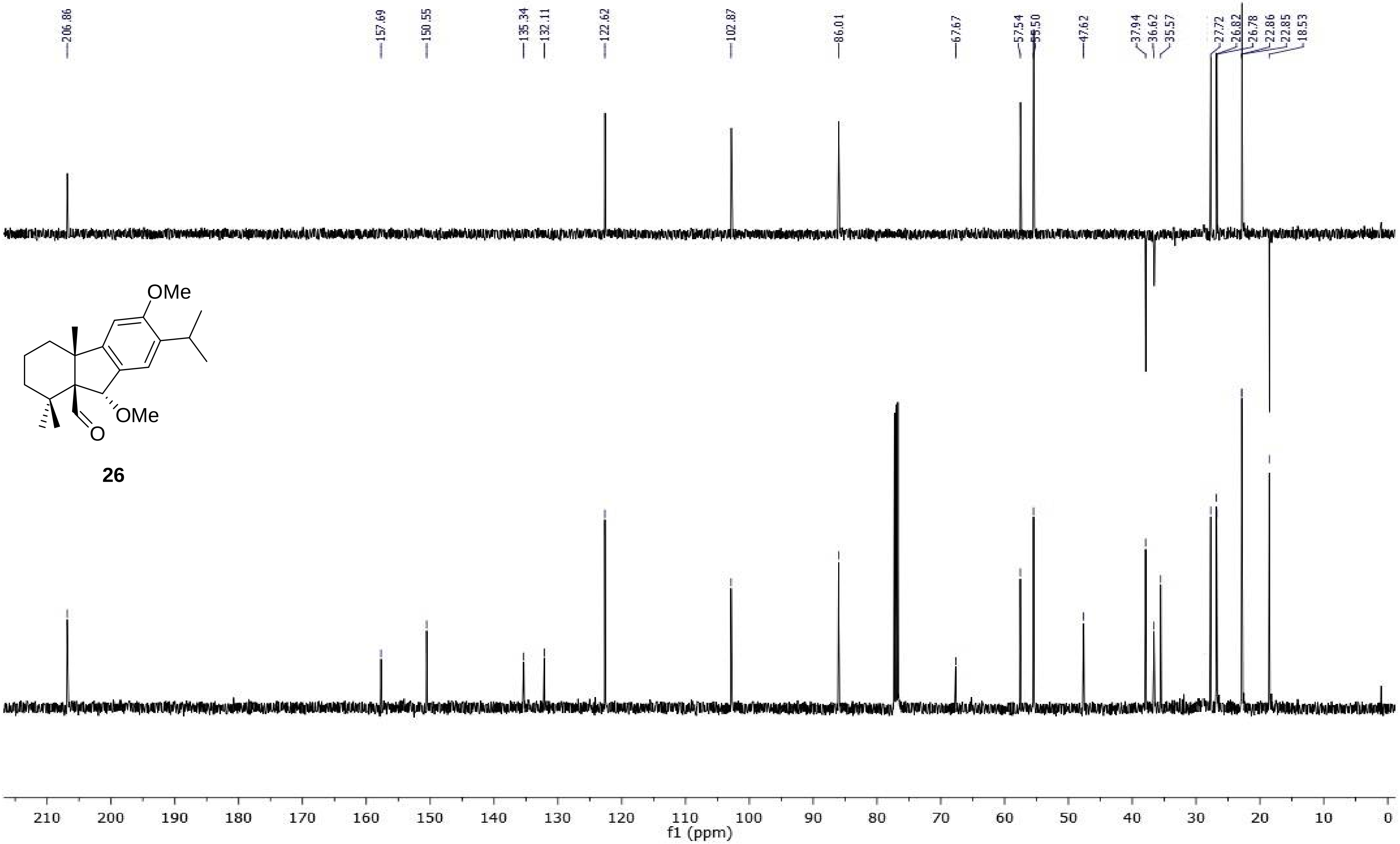
206.86
157.69
150.55
135.34
132.11
122.62
102.87
86.01
67.67
57.54
55.50
47.62
37.94
36.62
35.57
27.72
26.82
26.78
22.86
22.85
18.53
OMe
OMe
O
26
210 200 190 180 170 160 150 140 130 120 110 100 90 80 70 60 50 40 30 20 10 0
f1 (ppm)

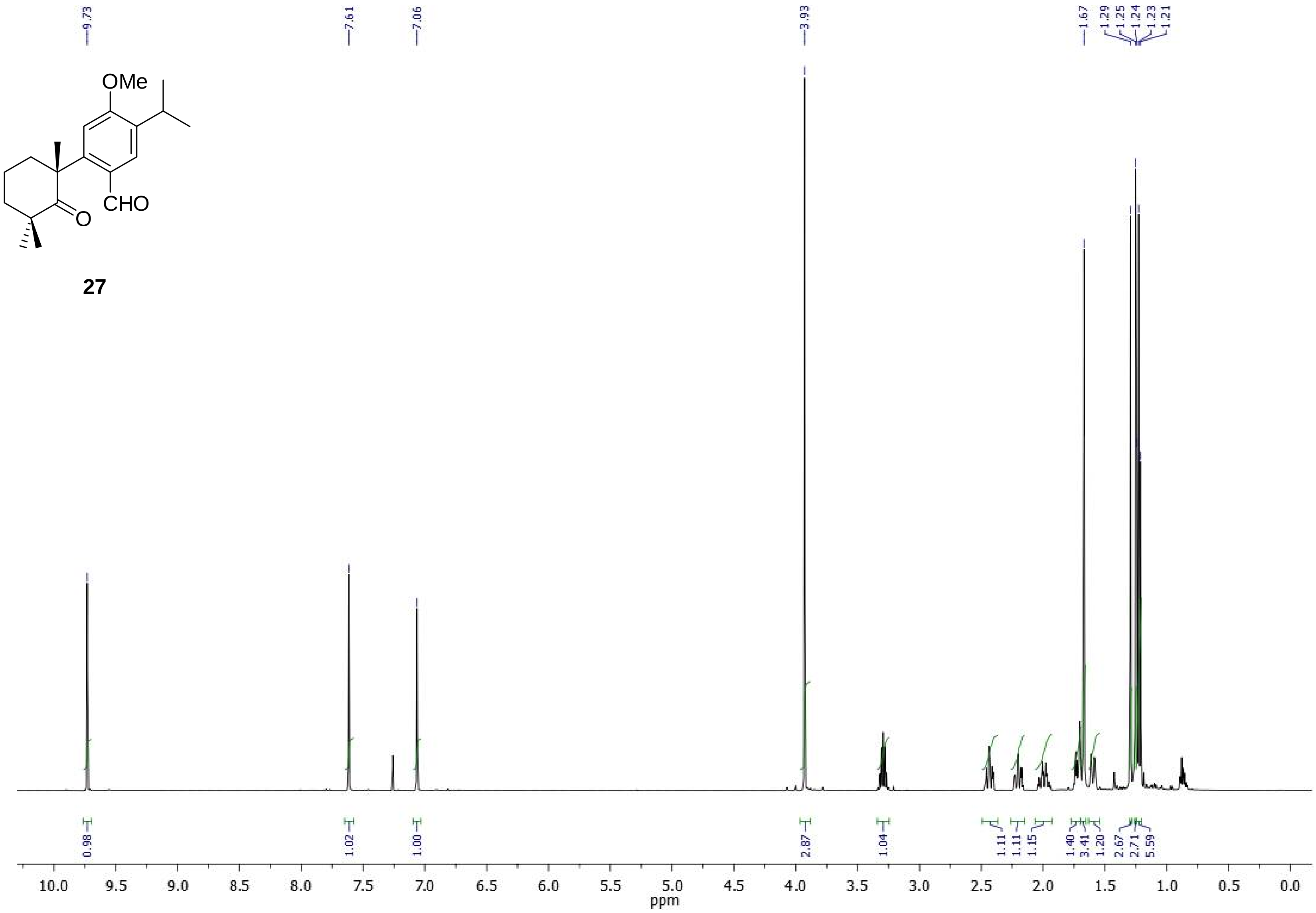
OMe
CHO
O
9.73
7.61
7.06
3.93
1.67
1.29
1.25
1.24
1.23
1.21
0.98
1.02
1.00
2.87
1.04
1.11
1.11
1.15
1.40
3.41
1.20
2.67
2.71
5.59
10.0 9.5 9.0 8.5 8.0 7.5 7.0 6.5 6.0 5.5 5.0 4.5 4.0 3.5 3.0 2.5 2.0 1.5 1.0 0.5 0.0
ppm

27

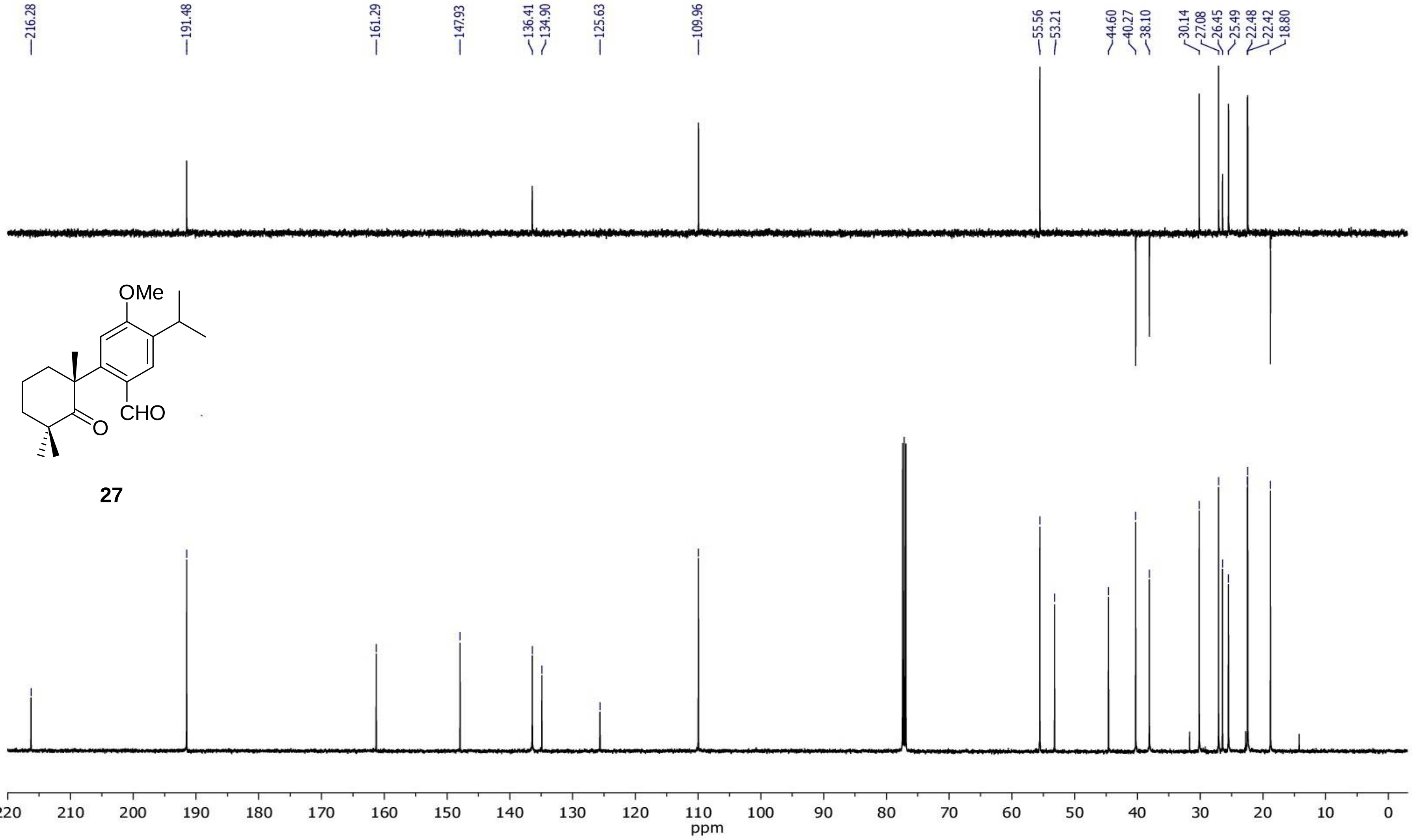
216.28
191.48
161.29
147.93
136.41
134.90
125.63
109.96
55.56
53.21
44.60
40.27
38.10
30.14
27.08
26.45
25.49
22.48
22.42
18.80
OMe
CHO
O
220 210 200 190 180 170 160 150 140 130 120 110 100 90 80 70 60 50 40 30 20 10 0
ppm

27

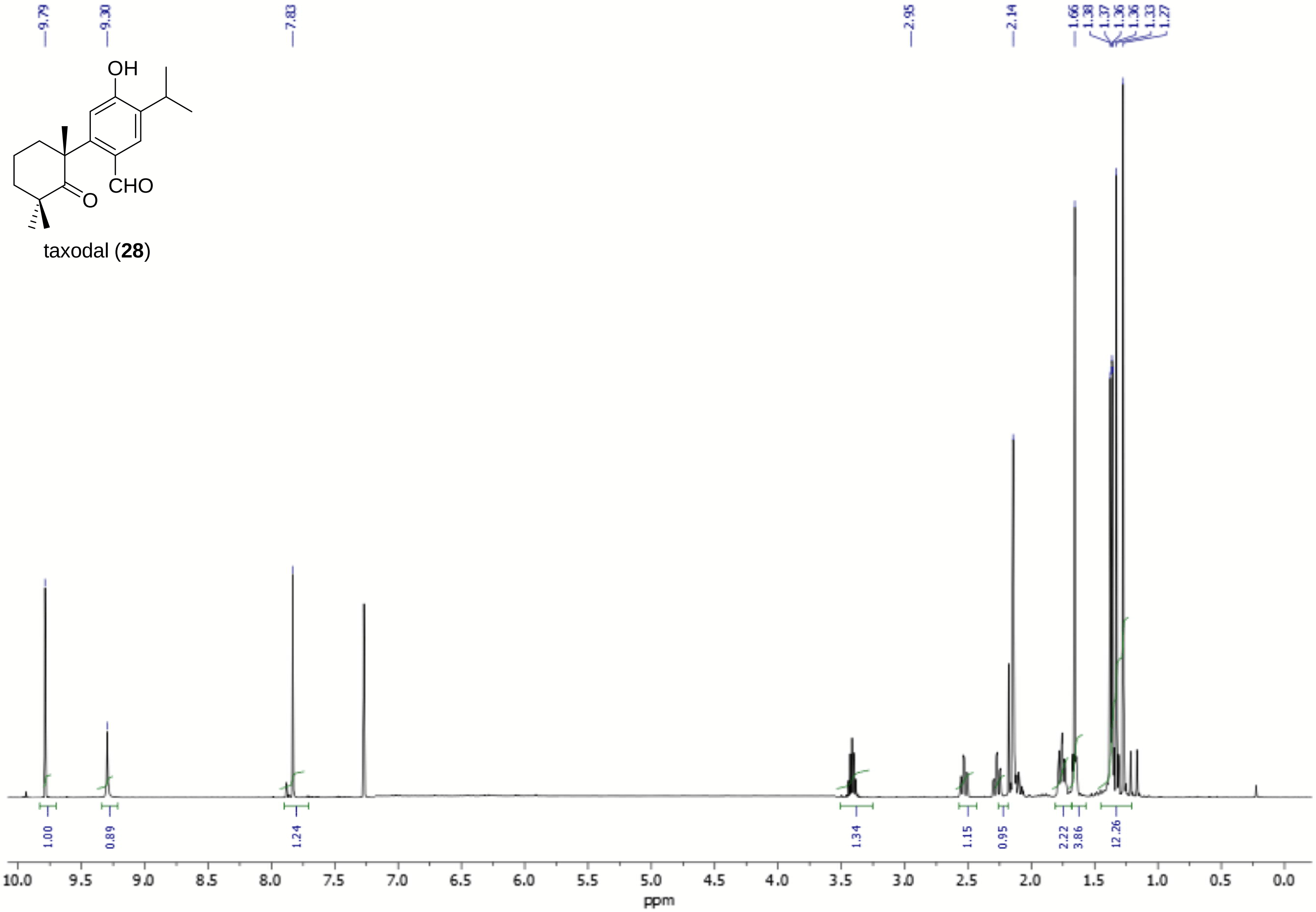

OH
CHO
O
9.79
9.30
7.83
2.95
2.14
1.66
1.38
1.37
1.36
1.36
1.33
1.27
1.00
0.89
1.24
1.34
1.15
0.95
2.22
3.86
12.26
10.0
9.5
9.0
8.5
8.0
7.5
7.0
6.5
6.0
5.5
5.0
4.5
4.0
3.5
3.0
2.5
2.0
1.5
1.0
0.5
0.0
ppm

taxodal (**28**)

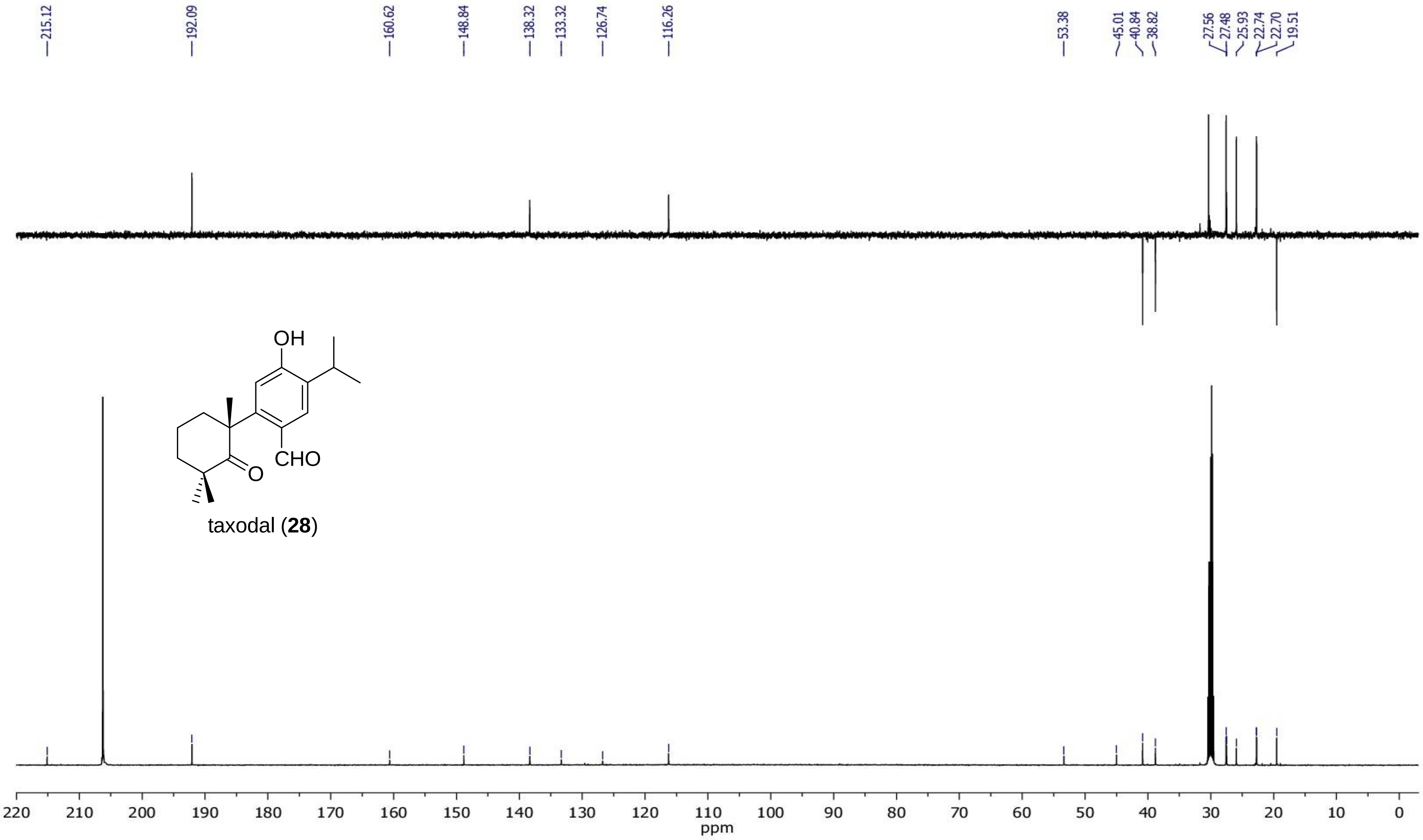
215.12
192.09
160.62
148.84
138.32
133.32
126.74
116.26
53.38
45.01
40.84
38.82
27.56
27.48
25.93
22.74
22.70
19.51
OH
CHO
O
taxodal (28)
220 210 200 190 180 170 160 150 140 130 120 110 100 90 80 70 60 50 40 30 20 10 0
ppm

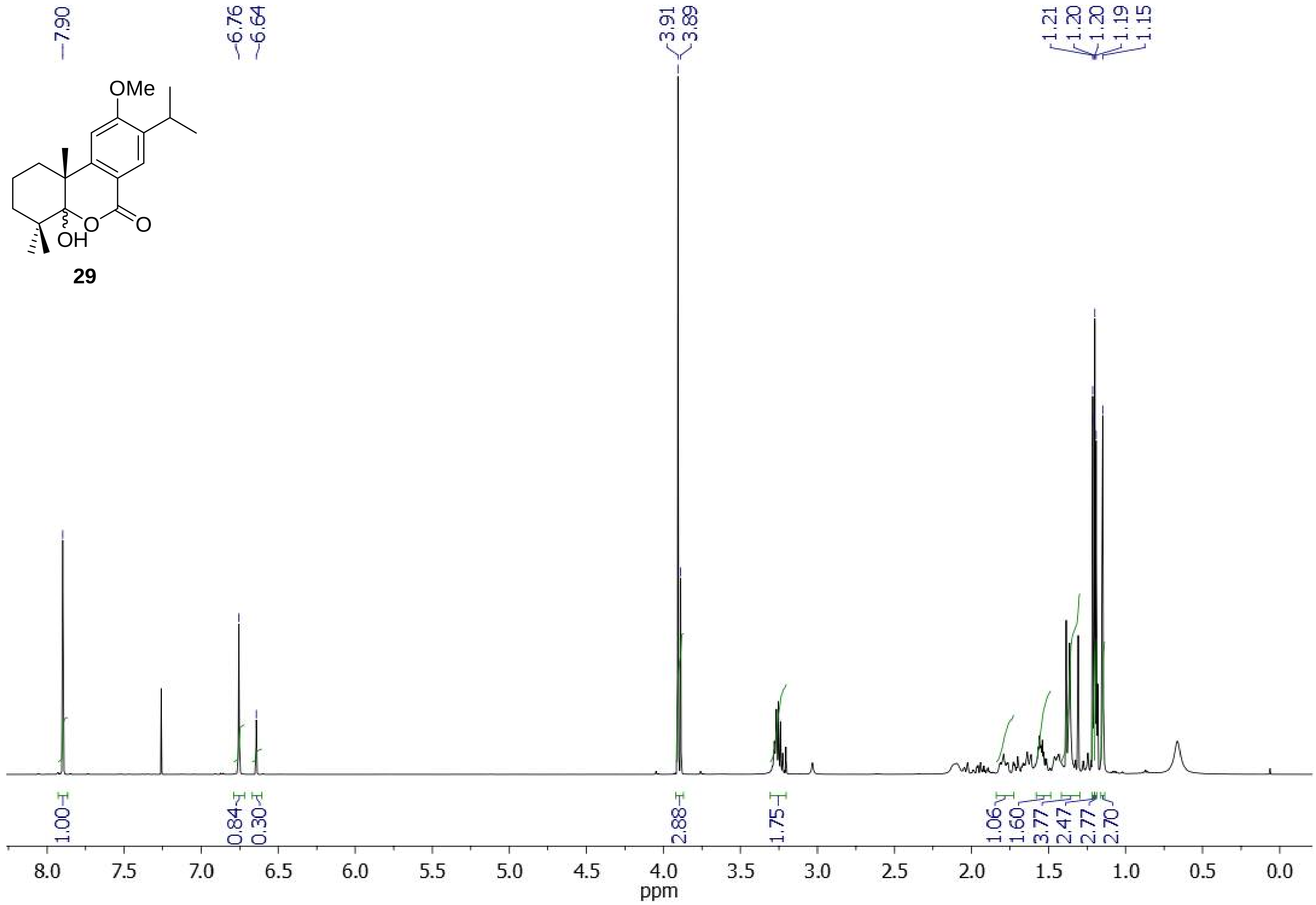

7.90
6.76
6.64
3.91
3.89
1.21
1.20
1.20
1.19
1.15
OMe
OH
O
O
29
1.00
0.84
0.30
2.88
1.75
1.06
1.60
3.77
2.47
2.77
2.70
8.0
7.5
7.0
6.5
6.0
5.5
5.0
4.5
4.0
3.5
3.0
2.5
2.0
1.5
1.0
0.5
0.0
ppm

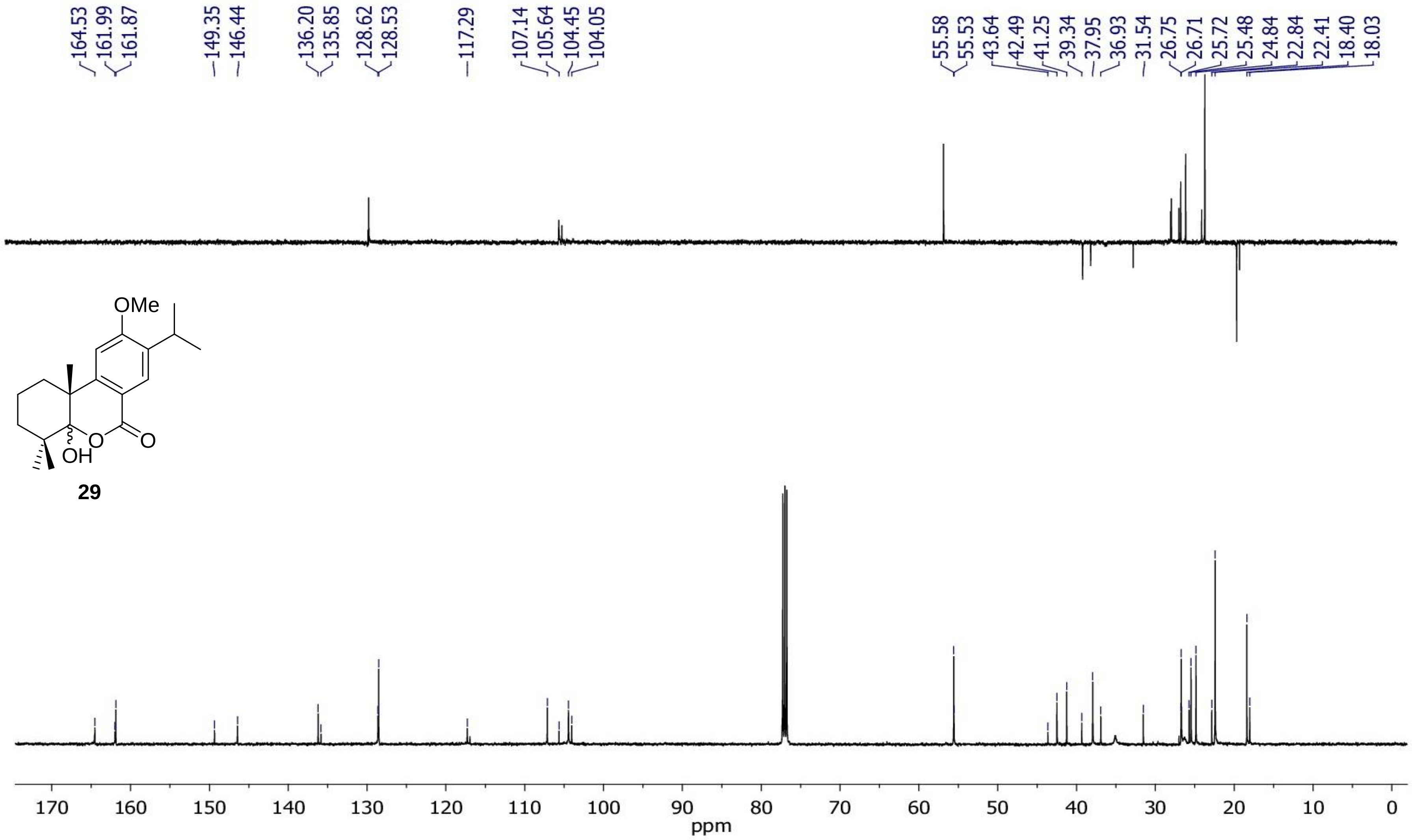
164.53
161.99
161.87
149.35
146.44
136.20
135.85
128.62
128.53
117.29
107.14
105.64
104.45
104.05
55.58
55.53
43.64
42.49
41.25
39.34
37.95
36.93
31.54
26.75
26.71
25.72
25.48
24.84
22.84
22.41
18.40
18.03
OMe
OH
O
O
29
170 160 150 140 130 120 110 100 90 80 70 60 50 40 30 20 10 0
ppm

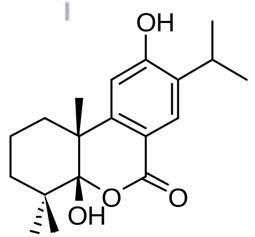

cupresol (**30**)

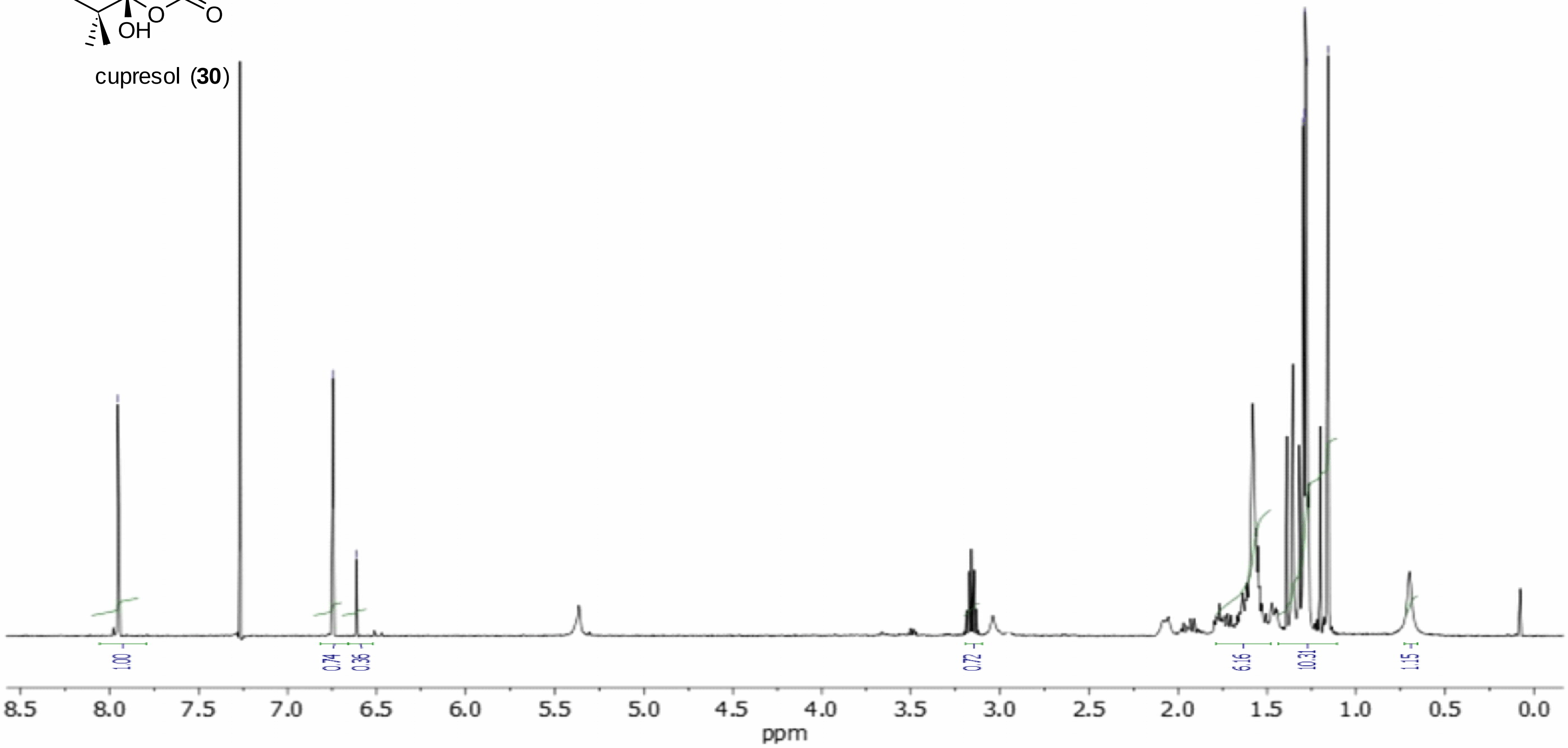

7.95
6.75
6.61
1.30
1.29
1.29
1.28
1.16
1.00
0.74
0.36
0.72
6.16
10.31
1.15
8.5
8.0
7.5
7.0
6.5
6.0
5.5
5.0
4.5
4.0
3.5
3.0
2.5
2.0
1.5
1.0
0.5
0.0
ppm

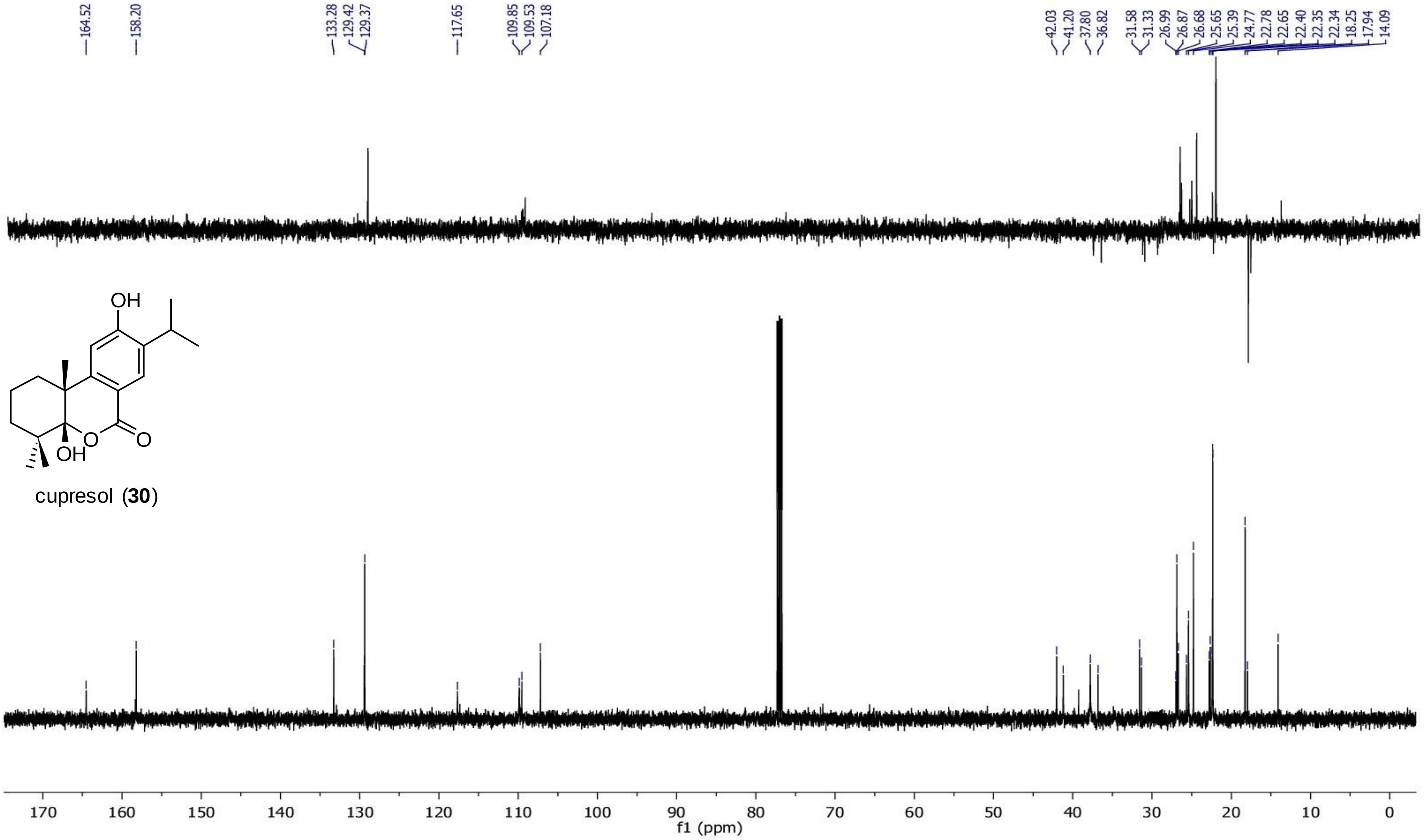
164.52
158.20
133.28
129.42
129.37
117.65
109.85
109.53
107.18
42.03
41.20
37.80
36.82
31.58
31.33
26.99
26.87
26.68
25.65
25.39
24.77
22.78
22.65
22.40
22.35
22.34
18.25
17.94
14.09
OH
OH
O
O
170 160 150 140 130 120 110 100 90 80 70 60 50 40 30 20 10 0
f1 (ppm)

cupresol (**30**)
